# Spondylosis deformans as an indicator of transport activities in archaeological dogs: A systematic evaluation of current methods for assessing archaeological specimens

**DOI:** 10.1371/journal.pone.0214575

**Published:** 2019-04-17

**Authors:** Katherine J. Latham, Robert J. Losey

**Affiliations:** Department of Anthropology, University of Alberta, Edmonton, Alberta, Canada; Seoul National University College of Medicine, REPUBLIC OF KOREA

## Abstract

Over the past several decades archaeologists have used the spinal pathology spondylosis deformans as an indicator that archaeological dogs were used to pull or carry loads. This interpretive approach is largely based upon observations of prehistoric dog remains and archaeologist’s interpretations of veterinary literature on recent sled dogs and other draft animals. However, no comparative large-scale studies of the occurrence of spondylosis deformans in wild canids, transport dogs, and dogs never involved in pulling or carrying loads have been published. To evaluate the reliability of spondylosis deformans in archaeological dogs as an indicator of participation in transport activities, 136 modern non-transport dogs, 19 sled dogs, and 241 wolves were systematically analyzed for the occurrence of spondylosis deformans. Our results indicate this pathology is not a reliable skeletal indicator of dog transport because the disease is prevalent in both dogs and wolves, regardless of their occupational histories. Numerous factors correlate with the occurrence and manifestation of this disease in canids, including age, body size, sex, and inbreeding. As such, it remains extremely challenging to identify specific etiologies for spondylosis deformans in archaeological specimens.

## Introduction

Historically, humans and dogs have engaged in a wide variety of working relationships. For example, dogs have pulled and carried loads in many parts of the world, including much of the circumpolar North, the Great Plains of North America, and southern Europe [1–9]. Given the widespread distribution of dog transport activities and their significant roles in some societies, there is reason to suspect that dogs were involved in similar working relationships with humans in the past. Unfortunately, these relationships are difficult to interpret archaeologically because there is little unambiguous artifactual evidence of dog transport. Further, many of the technologies involved (harnesses, sleds) were likely constructed of perishable materials and thus unlikely to be preserved in many archaeological settings.

The skeletal remains of dogs might hold clues as to their past involvement in transport as multiple authors have suggested [10–16]. If such signatures are unique to dogs involved in transport activities, then reliable inferences about the participation of dogs in transport activities might be made. The most commonly mentioned skeletal lesion used by archaeologists as evidence for dog involvement in transport is the spinal pathology spondylosis deformans [7, 10, 11, 13–18]. Spondylosis deformans is a degenerative spinal disease in which osteophytes form at the margins of vertebral bodies secondary to degeneration of the annulus fibrosus of the intervertebral disc [19–22]. Osteophytes can range in size from small spurs which do not extend beyond the vertebral endplate, to large scoop shaped growths extending toward the intervertebral disc, which in severe cases may ankylose with the adjacent vertebra to form a bony bridge over the intervertebral disc space [19, 20, 23, 24]. The rather conspicuous nature of this disease likely makes its presence obvious to many who work with archaeological skeletal remains.

Spondylosis deformans is relatively well documented in veterinary literature [20–22, 25–29] and has multiple potential causes. While the disease is degenerative, being correlated with aging in dogs and many other mammals, it can also be caused by mechanical stress, triggered by trauma and/or habitual activity [19, 20, 24]. These studies indicate that modern pet dogs are affected by high rates of spondylosis in general, and that factors such as body size and genetics can be linked to the development of the disease [20, 22, 25–29]. The disease has also been observed in foxes [30] and wolves and may occur at higher rates among inbred canid populations [31].

Several interrelated factors may account for the proposition in the archaeological literature that spondylosis deformans is an indicator of dog involvement in transport. First, spondylosis deformans was documented in sled dogs used by the British Antarctic Survey [32]. However, this study noted that the rate and pattern of spondylosis deformans observed was not unusual for dogs of advanced age—in other words, the cause(s) of the condition in these dogs was unclear [32]. Nonetheless, archaeologists have drawn upon this study to argue for a link between the presence of this disease in archaeological dog remains and the participation of past dogs in transport [10, 11, 13, 15, 16]. Later papers cited these earlier archaeological arguments to support similar interpretations of cases of spondylosis deformans in other ancient dogs [7, 14, 17, 18]. Second, the existing veterinary literature on spondylosis deformans in dogs largely focuses on the condition in specific breeds [25, 26, 29], differences in condition prevalence between breeds [27], or the etiology of the disease across a suite of affected and non-affected animals [20, 21, 28, 29]. Most importantly here, the Bellars and Godsal [32] paper is the only study in which transport dogs were part of the assessed individuals. In other words, with existing data it is very difficult to assess how and if spondylosis deformans manifests differently in transport versus non-transport dogs. Further, it is unclear how or if the disease differs between dogs and wild canids such as wolves, the latter perhaps being a better analog for free-ranging early dogs than modern pets, which are the product of selective breeding and are often highly sedentary. Further complicating the matter is that spondylosis deformans data in existing literature was gathered through a variety of methods, ranging from observations on skeletonized specimens, to those made on radiographs and autopsied animals. Such differences likely render results that differ in part solely due to collection methods.

To address these shortcomings, we present comparative data on the occurrence of spondylosis deformans in the skeletons of 19 modern sled dogs, 136 non-transport dogs, and 241 wolves from North America and Europe. The occurrence, distribution, and severity of lesions from this disease are presented for comparison between canid groups. Differences observed across groups and implications for the use of these lesions as skeletal indicators of dog transport in prehistory are then discussed.

## Materials

Vertebral columns and ribs from 396 dogs and wolves were assessed to evaluate the occurrence of spondylosis deformans in modern canids. The dog sample consisted of skeletal remains of 155 individuals curated at seven museums: 1) the University of Alberta Zooarchaeological Reference Collection, Edmonton, Canada; 2) the University of Alaska Museum of the North, Fairbanks, USA; 3) the Smithsonian Institution Museum of Natural History, Washington D.C., USA; 4) the California Academy of Sciences, San Francisco, USA; 5) the American Museum of Natural History, New York, USA; 6) the University of Nebraska State Museum, Lincoln, USA; 7) the Natural History Museum of Bern, Bern, Switzerland.

The domestic dog sample included both modern sled dogs (n = 19) and dogs that did not participate in load bearing or pulling activities (n = 125) as well as wild (non-captive) (n = 5) and captive (n = 6) dingoes (*Canis lupus dingo*) (a detailed breakdown of all groups is provided in S1 Table). Variable life history information such as breed and age at death was available for each specimen.

The non-transport dog sample (n = 125) included 106 dogs from 57 different breeds, as well as 19 individuals of unknown breed. Individuals of known breed were sub-categorized into three size groups following the American Kennel Club and Federation Cynologique Internationale standards for each breed [33, 34]. These size groups included 29 small dogs, 32 medium dogs, and 45 large dogs. A detailed list of breed information is available in S2 Table.

A total of 241 North American and Scandinavian gray wolves were assessed using specimens in five different museum collections (S3 Table): 1) the Royal Alberta Museum, Edmonton, Canada; 2) the University of Alaska Museum of the North, Fairbanks, USA; 3) the Smithsonian Institution Division of Mammals, Washington D.C., USA; 4) the California Academy of Sciences, San Francisco, USA; 5) the Swedish Museum of Natural History, Stockholm, Sweden.

The wolf sample included both wild (non-captive) (n = 228) and captive (n = 13) individuals (see S4 Table). Among this sample were individuals from a population of highly inbred wolves from Sweden (n = 81) where gray wolf populations were severely reduced in the mid-20^th^ century. Less than 10 individuals were present by the early 1970s when they were given legal protection by the Swedish government. In 1983 a single mating pair re-established the modern population present in Sweden today. The population has low genetic variability due to high rates of inbreeding [35].

Little life history information is available for the wolf sample but select data are available for some individuals (see S5 Table). Age of death information for the Swedish population was determined by the museum through assessment of dental cementum bands. For the Alaskan population, juvenile or adult status at death was estimated based upon epiphyseal fusion.

Some individuals of known age were present in all three analytic groups and used to make age-based assessments. Both dog samples were represented by an older population than the wolf sample, and these differences in age-profiles should be noted. It is unclear how the age profiles of the canid groups of unknown age differed, though it seems likely that wolves living in the wild rarely reached the advanced ages seen in domestic dogs.

## Methods

**Fig 1:**
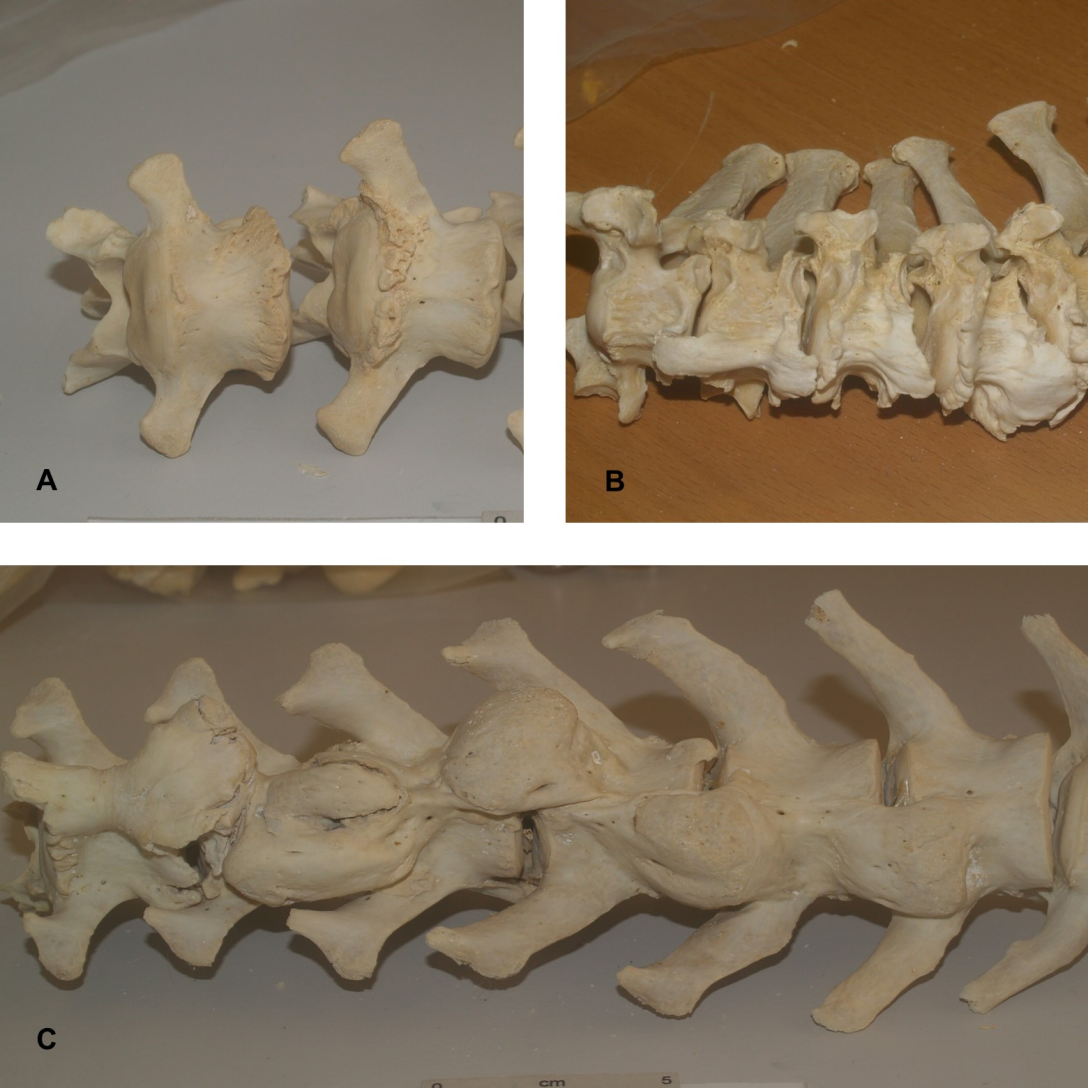
Examples of osteophyte grades. (A) Grade 1 osteophytes. (B) Grade 2 osteophytes. (C) Grade 3 osteophytes.

The presence of spondylosis deformans was evaluated using methods developed by osteologists and veterinarians [20–22, 25, 28, 29, 36, 37]. For all specimens, each vertebrae of the spinal column excluding the three sacral and ~20 caudal vertebrae were assessed. The presence or absence of spondylosis was recorded for both the cranial and caudal surfaces. Where spondylosis was present, the severity of the growth was numerically classified using Carnier et al’s [25] scoring system quoted below, with examples illustrated in Fig 1:

Grade 1 = small osteophytes placed on the edge of the epiphysis [endplate] were observed, but did not exceed the vertebral edge
Grade 2 = osteophytes were enlarged beyond the edge of the epiphysis [endplate], but did not connect to osteophytes on the opposite vertebra
Grade 3 = osteophytes placed on adjoining vertebrae connected one to each other, thus establishing an appreciable bony spur

Statistical analyses were performed to compare frequencies of spondylosis deformans between the different study groups, as well as the frequencies of various manifestations of this pathology within individual groups. Frequency comparisons were made using the Pearson’s Chi-squared Test for larger sample sizes and Fisher’s Exact Test for smaller sample sizes of five individuals or less. All statistical analyses were performed using Microsoft Excel 2016.

## Results

Spondylosis deformans was prevalent in dogs, regardless of their use in transport activities (Table 1). Non-transport dogs were most affected with 66.18% of individuals displaying these lesions. This rate increased (68.80%) when the small sample of dingoes was excluded. Non-transport dogs were affected with spondylosis deformans at a slightly higher frequency than sled dogs (66.18% vs. 63.16%, respectively), but the differences between the two groups were not significant (X^2^ = 0.08, p = 0.6231). Wolves were affected with spondylosis deformans at a significantly lower rate (21.16%) than both non-transport dogs (X^2^ = 49.9007, p = <0.0001) and sled dogs (X^2^ = 12.8420, p = <0.0001)(Table 2). Though just over 20% of all wolves were affected by spondylosis deformans, captive wolves had a significantly higher incidence of the disease (76.92% vs. 17.98%, respectively) than their wild counterparts (p = <0.0001). The Swedish population of inbred wolves (27.16%) was affected by the disease at higher rates than the non-inbred wolves from all other regions (18.13%), though not at a statistically significant level (X^2^ = 2.07, p = 0.1048). There were no significant sex-based differences in the frequencies at which individuals were affected by spondylosis deformans in any of the canid groups.

**Table 1:** Spondylosis deformans in dogs by percentage of individuals affected.

	All Non-transport dogs	Dogs w/o Dingoes	All Dingoes	Captive Dingoes	Wild Dingoes	Sled Dogs
	(n = 136)	(n = 125)	(n = 11)	(n = 6)	(n = 5)	(n = 19)
	n (%)	n (%)	n (%)	n (%)	n (%)	n (%)
**Female**	42/57(73.68)	42/54(77.78)	0/3(0.00)	0/1(0.00)	0/2(0.00)	3/4(75.00)
**Male**	42/62(67.74)	39/55(70.91)	3/7(42.86)	3/5(60.00)	0/2(0.00)	7/7(100.00)
**Unknown**	6/17(35.29)	5/16(31.25)	1/1(100.00)	0/0(0.00)	1/1(100.00)	2/8(25.00)
**Adult**	90/130(69.23)	86/121(71.07)	4/9(44.44)	3/5(60.00)	1/4(25.00)	12/19(63.16)
**Juvenile**	0/6(0.00)	0/4(0.00)	0/2(0.00)	0/1(0.00)	0/1(0.00)	0/0(0.00)
**Total**	**90(66.18)**	**86(68.80)**	**4(36.36)**	**3(50.00)**	**1(20.00)**	**12(63.16)**

**Table 2:** Spondylosis deformans in wolves by percentage of individuals affected.

	All Wolves	Wild Wolves	Captive Wolves	Inbred Wolves	Non-inbred Wolves
	(n = 241)	(n = 228)	(n = 13)	(n = 81)	(n = 160)
	n (%)	n (%)	n (%)	n (%)	n (%)
**Female**	17/103(16.50)	13/99(13.13)	4/4(100.00)	9/35(25.71)	8/68(11.76)
**Male**	30/124(24.19)	25/117(21.37)	5/7(71.43)	12/44(27.27)	18/80(22.5)
**Unknown**	4/14(28.57)	3/12(25.00)	1/2(50.00)	1/2(50.00)	3/12(25.00)
**Adult**	51/167(30.54)	41/155(26.45)	10/12(83.33)	22/58(37.93)	29/109(26.61)
**Juvenile**	0/74(0.00)	0/73(0.00)	0/1(0.00)	0/23(0.00)	0/51(0.00)
**Total**	**51(21.16)**	**41(17.98)**	**10(76.92)**	**22(27.16)**	**29(18.13)**

### Age

**Fig 2:**
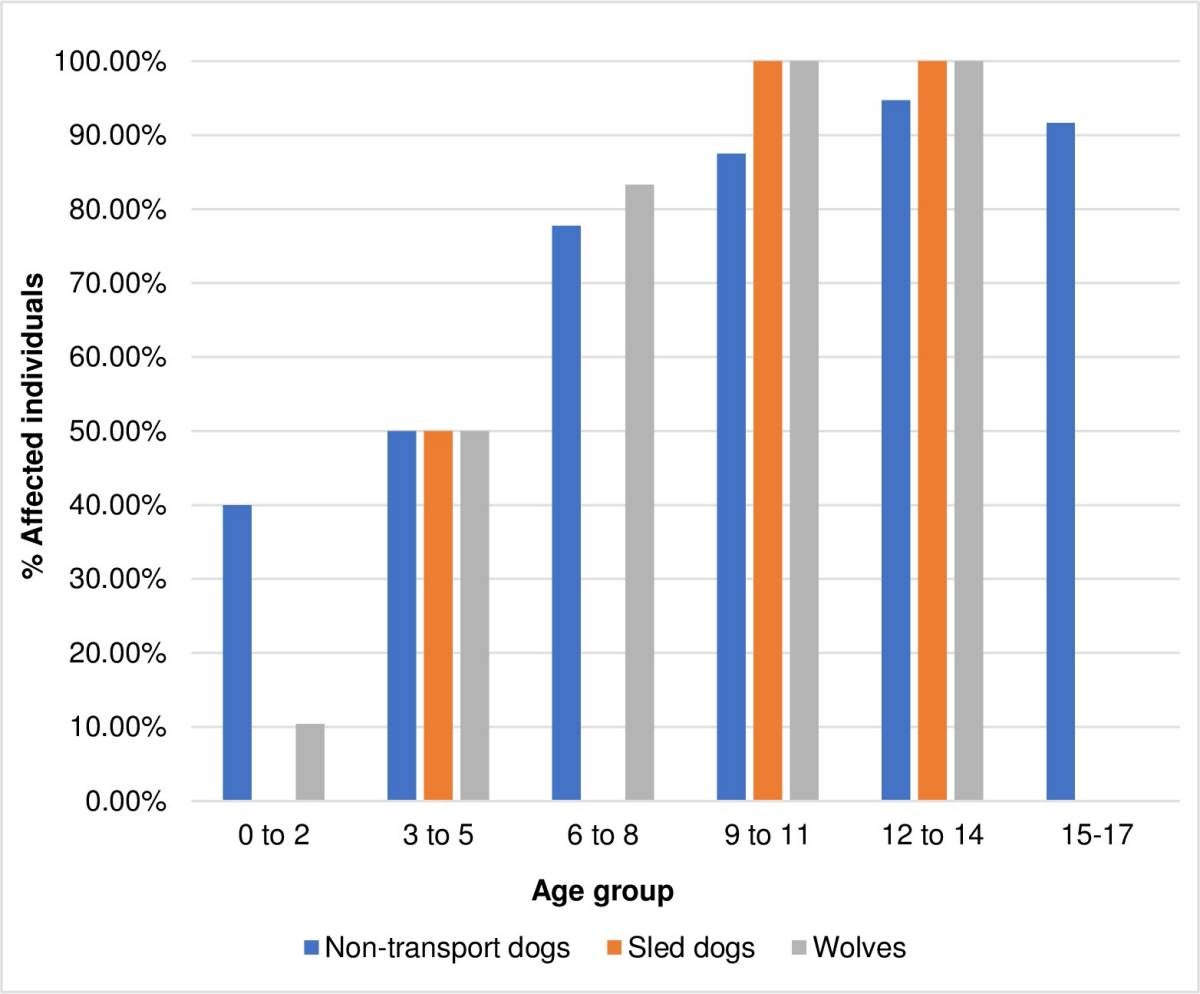
Spondylosis deformans in dogs and wolves of known age groups.

Spondylosis deformans was only observed in physically mature individuals—no juvenile individuals were affected in any analytic group (Tables 1 and 2). It is important to note that the number of juvenile non-transport dogs available for study was low and no juvenile sled dogs were available to assess. Age group data are presented in Table 3 and Fig 2. When individuals of known age are separated into age cohorts, a consistent increase in the frequency of spondylosis occurs with age in all groups (non-transport dogs, sled dogs, and wolves). This pattern is also apparent when using broader age categories. When non-transport dogs were compared to wolves, the frequency of affected individuals under five years of age and those six years of age or older differed significantly (non-transport dogs, X^2^ = 2.43, p = 0.0003; wolves, X^2^ = 15.41, p = <0.0001).

**Table 3:** Spondylosis deformans in dogs and wolves by age group and percentage of individuals affected.

Age range (years)	Non-transport dogs	Sled dogs	Wolves
	(n = 77)	(n = 10)	(n = 70)
	n (%)	n (%)	n (%)
**0–2**	2/5(40.00)	N/A	5/48(10.42)
**3–5**	4/8(50.00)	1/2(50.00)	6/12(50.00)
**6–8**	7/9(77.78)	N/A	5/6(83.33)
**9–11**	21/24(87.50)	6/6(100.00)	3/3(100.00)
**12–14**	18/19(94.74)	2/2(100.00)	1/1(100.00)^a^
**15–17**	11/12(91.67)	N/A	N/A
**Total**	**63(81.82)**	**9(90.00)**	**20(28.57)**

^**a**^ captive individual

### Size-based breed cohorts

Non-transport dogs were also assessed by size-based breed cohorts. The proportion of individuals affected by spondylosis deformans increased with body size: small dogs 55.17%, medium dogs 65.12%, and large dogs 91.11%. The difference between small dogs and medium dogs was not statistically significant (X^2^ = 0.28, p = 0.3959), but it was between small and large breeds (p = 0.0005) and between medium and large breeds (p = 0.0040).

### Number and proportion of affected endplates

The total number of endplates with osteophytes varied greatly among affected individuals in all groups. Non-transport dogs were found to have anywhere between one and 44 endplates affected by spondylosis deformans with an average of 16.31 per individual. Sled dogs had fewer osteophytes than non-transport dogs with a range of one to 27 endplates affected per individual (averaging 8.08). Individual wolves had anywhere between one and 45 affected endplates, a range similar to non-transport dogs. However, the average number of affected endplates per individual wolf was 8.80 endplates, much lower than in non-transport dogs.

Non-transport dogs had a significantly higher proportion of endplates with spondylosis deformans (1468/7334 (20.02%)) when compared to the other groups (non-transport dogs versus sled dogs, X^2^ = 7.87, p = 0.0017; non-transport dogs versus wolves, X^2^ = 1361.49, p = <0.0001). Sled dogs had a lower proportion of affected endplates (163/1026 (15.89%)) than non-transport dogs but were significantly more affected than wolves (sled dogs versus wolves, X^2^ = 336.13, p = <0.0001). Wolves had the lowest number of affected endplates (449/12981 (3.46%)). When wolves were assessed separately, captive wolves (180/700 (25.71%)) had a significantly higher proportion of affected vertebrae than their wild counterparts (269/12281 (2.19%) (X^2^ = 1059.51, p = <0.0001)) or any other canid group. Differences between the captive wolves and the non-transport dogs (X^2^ = 10.11, p = 0.0004) and between the captive wolves and sled dogs (X^2^ = 20.22, p = <0.0001) were also significant.

### Distribution of osteophytes

**Fig 3:**
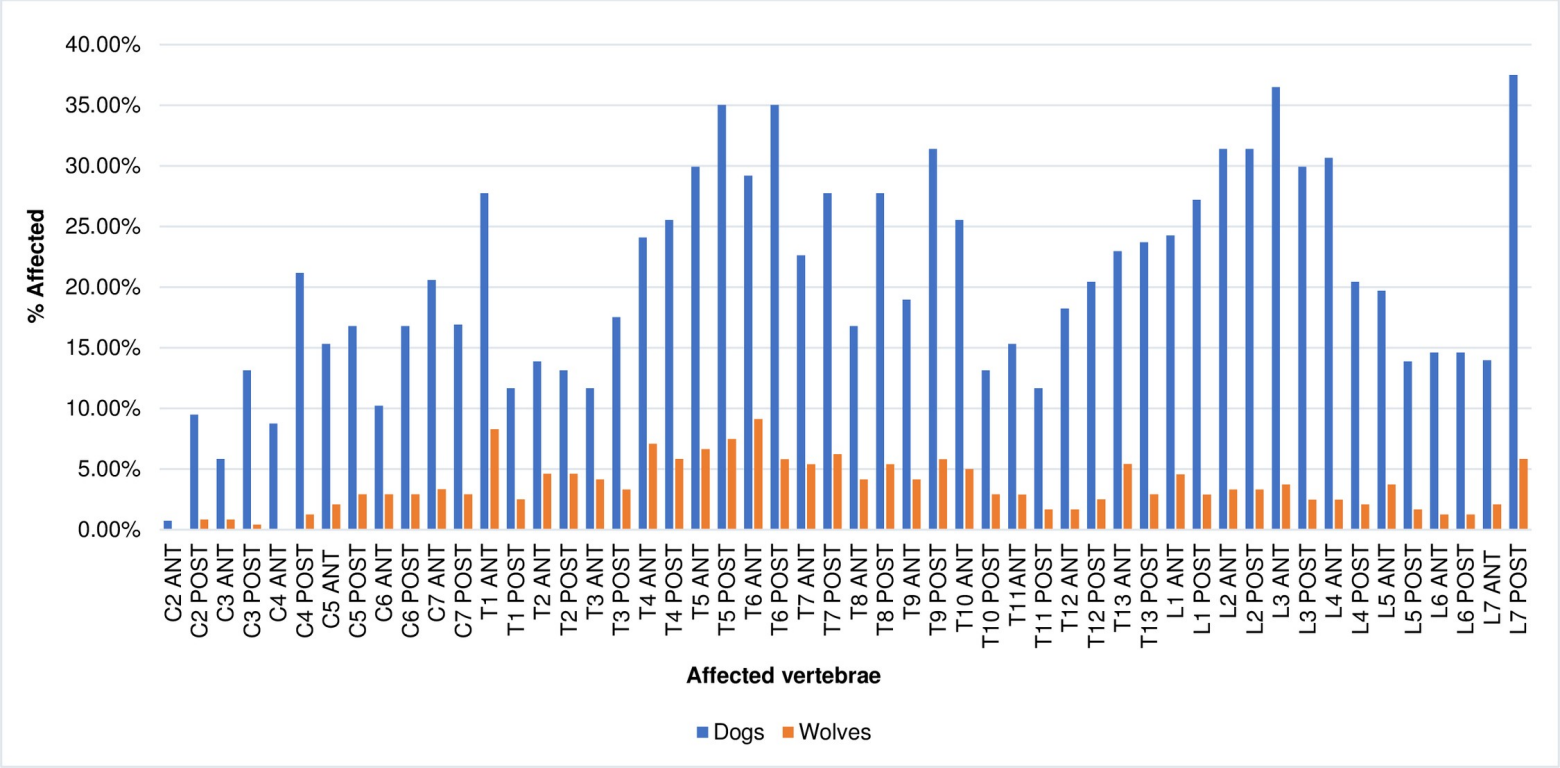
Distribution of osteophytes in dogs and wolves by percentage of endplates affected.

**Fig 4:**
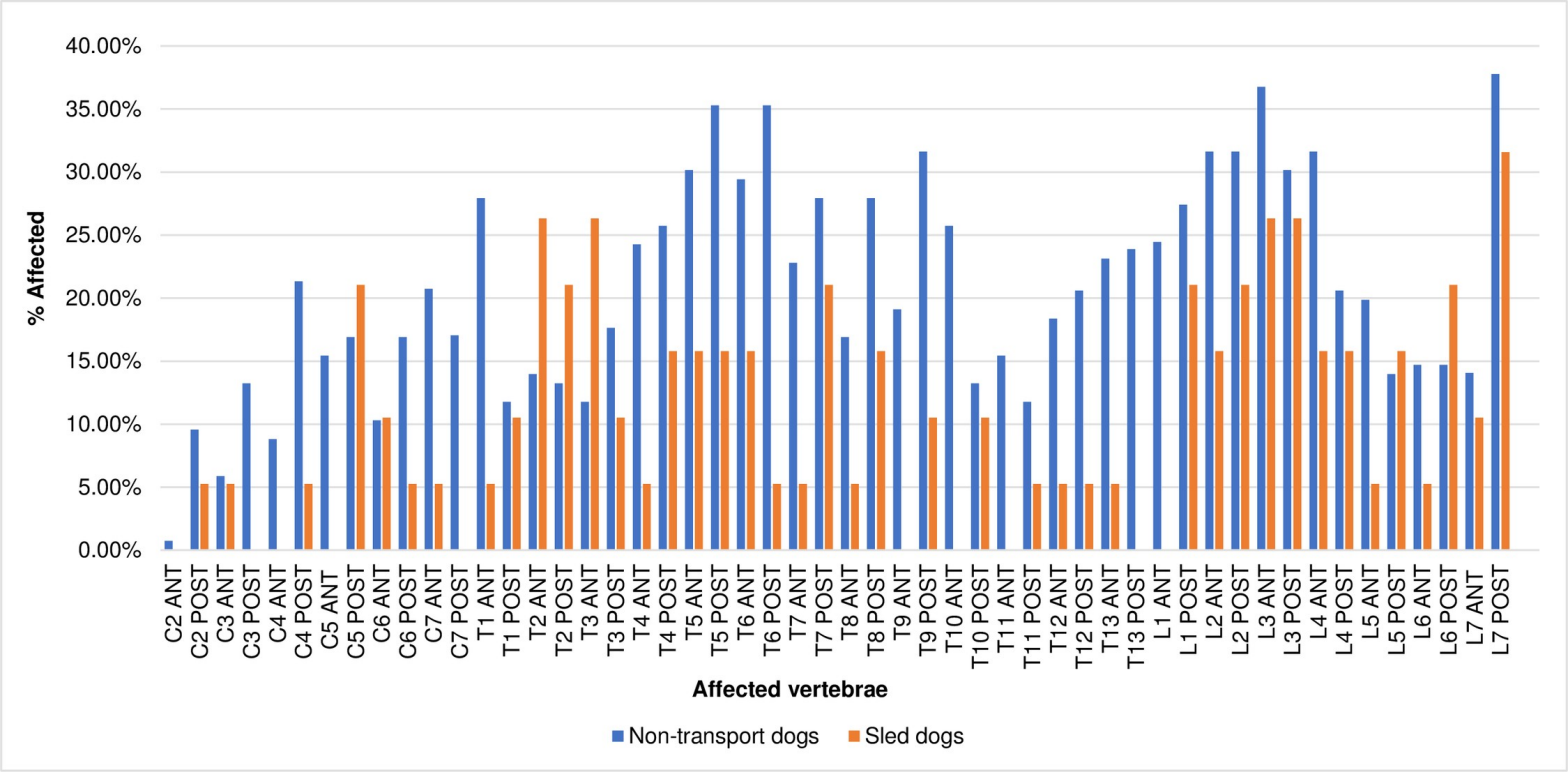
Distribution of osteophytes in dogs by percentage of endplates affected.

The distribution of affected endplates by region of the spine is shown in Figs 3 and 4. Non-transport dogs were least affected by marginal osteophytes in the cervical region of the spine and most affected in the lumbar region. Peaks in frequency occurred at the T1 cranial, T5 caudal, T6 caudal, and T9 caudal endplates as well as the cranial L3 and caudal L7 endplates (Figs 3 and 4). While the rate at which cervical vertebrae were affected differed from the other regions significantly (cervical versus thoracic, X^2^ = 80.49, p = >0.0001; cervical vs. lumbar, X^2^ = 82.29, p = <0.0001), the difference between thoracic and lumbar vertebrae was not significant (X^2^ = 0.78, p = 0.3148).

Sled dogs followed the same pattern, with the cervical vertebrae least affected and lumbar vertebrae most affected. Peaks occurred at the T2 and T3 cranial endplates and the cranial and caudal L3 and L7 caudal endplates (Fig 4). In this group, the rates at which all three regions were affected differed significantly (cervical versus thoracic, X^2^ = 7.72, p = 0.0038; cervical versus lumbar, X^2^ = 19.8, p = <0.0001; thoracic versus lumbar, X^2^ = 5.76, p = 0.010).

Among wolves the cervical region was also least affected, but in contrast with the dogs, they were most affected by spondylosis deformans in the thoracic region. Peaks occurred at the T1 cranial and T6 cranial endplates and the L1 cranial and L7 caudal endplates (Fig 3). As with the sled dogs, the rates at which all three regions were affected differed significantly (cervical versus thoracic, X^2^ = 68.00, p = <0.0001; cervical versus lumbar, X^2^ = 16.13, p = <0.0001; thoracic versus lumbar, X^2^ = 19.53, p = <0.0001).

**Fig 5:**
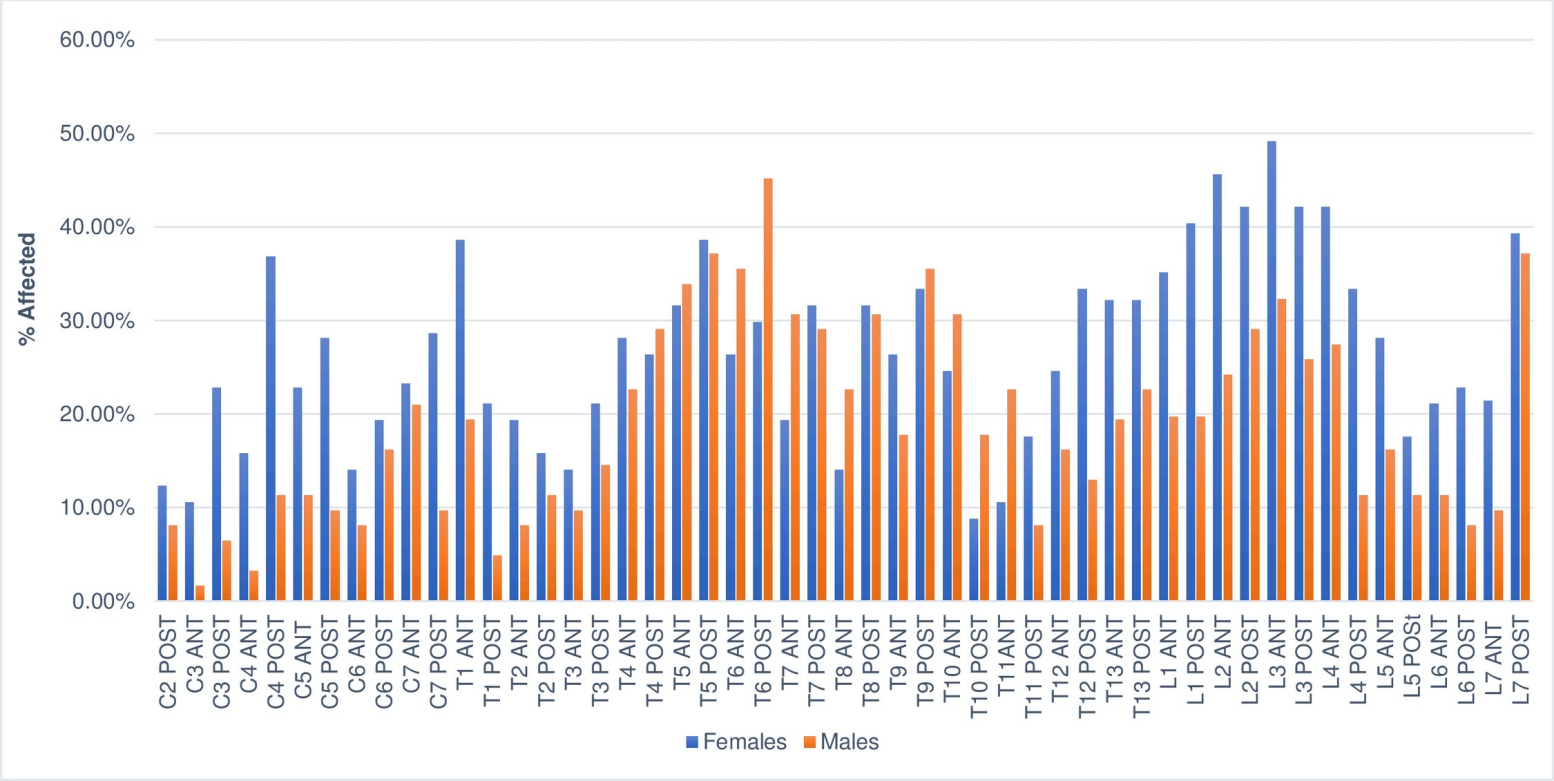
Distribution of osteophytes in non-transport dogs by sex.

Sex-based differences in the distribution of osteophytes were observed in non-transport dogs. Females had a higher prevalence (134/796 (16.83%)) of affected cervical endplates than males (66/686 (7.60%)). The difference between the two groups was highly significant (X^2^ = 29.43, p = <0.0001). Males and females were similarly affected in the thoracic region but again differed in the distribution of osteophytes in the lumbar region: females were affected at a rate of 273/796 (34.30%) and the frequency in males was 175/866 (20.21%). The difference between the two groups was highly significant (X^2^ = 30.54, p = <0.0001). Visual comparison of the distribution of osteophytes in female and male non-transport dogs is illustrated in Fig 5. There were no significant differences in the distribution of osteophytes by sex in wolves, and the sled dog population was too small to provide meaningful results.

### Grades of osteophytes

The severity of osteophyte formation for each group is presented in Table 4. In all groups, grade 1 osteophytes were most common, and grade 3 osteophytes were least common. The average grade of affected endplates for non-transport dogs was 1.35. In general, severe osteophytes were more likely to occur in the lower thoracic and lumbar vertebrae. The distribution of osteophyte grades in non-transport dogs is illustrated in S1 Fig. No sled dogs were affected with grade 3 osteophytes, and the average osteophyte grade for this group was 1.21. The distribution of osteophyte grades for sled dogs is illustrated in S2 Fig. Wolves were affected with an average osteophyte grade of 1.38. The occurrence of grade 3 osteophytes was limited to the lower thoracic and lumbar vertebral regions. The distribution of osteophytes by vertebrae in wolves is illustrated in S3 Fig.

**Table 4:** Severity of spondylosis deformans in affected endplates of dogs and wolves.

	Non-transport dogs	Sled dogs	Wolves
	n (%)	n (%)	n (%)
**Grade 1**	985(67.14)	86(52.76)	296(65.92)
**Grade 2**	450(30.67)	19(11.66)	137(30.51)
**Grade 3**	33(2.25)	0(0.00)	16(3.56)
**Average grade**	1.35	1.21	1.38

In general, the occurrence of osteophyte grades 1 and 2 increased with age across all groups. Stage 3 osteophytes were relatively rare in all groups and did not seem to be linked to age. In non-transport dogs, there was a substantial increase in the occurrence of grade 1 and grade 2 osteophytes after 8 years of age (S6 Table). These patterns are illustrated in S4 & S5 Figs.

The total number of sled dogs assessed by age was very small, but the individuals analyzed followed a pattern similar to the non-transport dogs. The overall number of osteophytes increased markedly after 11 years of age, but increased age was not necessarily related to the severity of the osteophytes (see S7 Table and S6 & S7 Figs).

Among wolves there was a steady increase in the total number of osteophytes as age increased and a marked increase in the occurrence of osteophytes after 8 years of age (S8 Table and S8 & S9 Figs). As with the dogs, grade 1 osteophytes were consistently the most numerous and again, increased age did not appear to be a predictor of severity.

## Discussion

**Fig 6:**
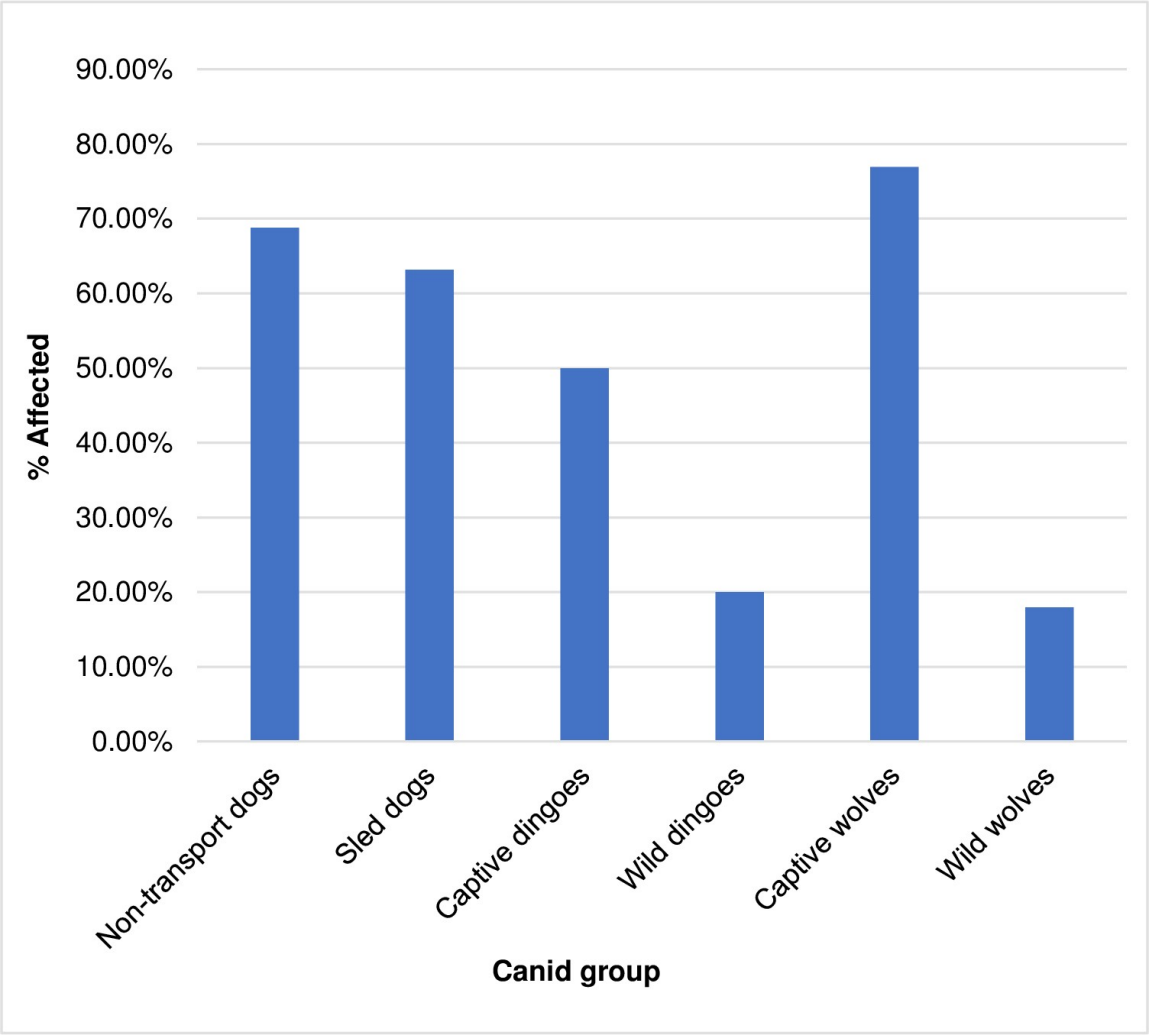
Prevalence of spondylosis deformans in all canid groups.

Spondylosis deformans was prevalent in dogs regardless of their participation in transport activities (Fig 6). While non-transport dogs had the highest proportion of affected individuals, they did not differ significantly from sled dogs in this regard.

In general, wolves were less affected by spondylosis deformans than either of the dog groups (Fig 6). Overall, 21.16% of wolves were affected with the disease, but wolves living in captivity were more affected than any other canid group in the study, with 76.92% of individuals exhibiting the lesions. This differs drastically from the frequency observed in wild wolves (19.98%) and is highly significant. The prevalence in captive wolves was similar to dogs, suggesting that the environment in which a canid lives contributes to its likelihood of developing the disease, a point we return to below. Though the sample size was very small, dingoes also demonstrated this pattern, with 50.00% of captive dingoes affected with spondylosis compared to 20.00% of the wild population.

The prevalence of spondylosis deformans in non-transport dogs and sled dogs was similar to that observed in previous studies. Using radiographs Morgan [20] found that 71 of 116 (61%) individuals from various breeds were affected by the disease. In a study of 140 skeletonized dogs from England, Read and Smith [27] documented that 75% were affected. Finally, a study of radiographs of dogs from England, the United States, and Sweden found that 110 of 175 (62.8%) individuals were affected [22].

The prevalence of spondylosis deformans observed in both non-transport and sled dogs is comparable to that observed among the British Antarctic Survey’s sled dogs. Bellars and Godsal [32] reported that 9/11 (81.82%) of their radiographed sleds dogs were affected. While the frequency observed in the Antarctic sled dogs was higher than those observed in the non-transport or sled dogs assessed in the present study, the differences between the groups are not significant (versus this study’s non-transport dogs, X^2^ = 0.35, p = 0.2825; versus this study’s sled dogs, X^2^ = 0.35, p = 0.2825), and are likely the product of a comparatively small sample consisting exclusively of older dogs. In fact, when dogs from the present study of the same age range as the Antarctic dogs (ages 5 to 10 years) are considered, the frequency of individuals affected with spondylosis deformans is nearly identical to that of the Antarctic dogs (31/38: 81.58%).

The proportion of individuals with spondylosis deformans steadily increased with age in all analytic groups (Fig 2). This trend was not surprising since spondylosis is a degenerative disease, and other studies have shown a similar correlation in dogs [20, 22, 25, 27, 29] and other mammal species, including humans [19, 23, 24, 30, 38–42].

This correlation may also explain the higher prevalence of spondylosis deformans among dogs and captive wolves as compared to wild wolves. In the wild, few wolves live beyond four or five years of age [43] though in rare cases they can reach up to 15 years of age [44]. Removed from the dangers of the wild, wolves living in captivity are more likely to survive to old age and can live up to 17 years [44]. For domestic dogs living in the care of humans, the average lifespan is 10–13 years [45, 46] though they too can live much longer. The present study found that across canid groups, half of all individuals aged 3–5 years were already affected with the disease and that the proportion of individuals affected steadily increased after 5 years of age. Assuming captivity can extend the lives of wolves, one would expect the disease to be more common among the captive population than among wild individuals.

From an archaeological perspective, these observations suggest that, rather than an indicator of participation in transport activities, higher rates of spondylosis deformans in a population of archaeological dogs could indicate the presence of older individuals. A high frequency of affected vertebrae may further support such a conclusion. Furthermore, high frequency of osteophytes in archaeological canids may indicate some degree of human care and provisioning, as canids are less likely to reach advanced ages when living in the wild.

Osteophyte severity was assessed in all canid groups. Though no meaningful age-related patterns were observed, we recommend that researchers documenting the occurrence of spondylosis deformans in canid specimens continue to evaluate the severity of osteophytes. Grade 3 osteophytes, though rare, are large and conspicuous and therefore more likely to be observed by archaeologists, especially those without training in paleopathology. In contrast, grade 1 osteophytes, which are most common in canids, are smaller, less conspicuous, and more likely to be overlooked. By systematically describing the severity of osteophytes on all vertebrae, misrepresentation of disease prevalence within an archaeological assemblage can be avoided.

Previous studies of domestic dogs [20, 22, 25, 26, 29] argue that certain breeds may be genetically predisposed to developing spondylosis deformans. In the present study, inbred wolves were found to have a higher frequency of the disease than their non-inbred counterparts. Though the two populations did not differ significantly in frequency, there is still a relatively high degree of probability (~90%) that these differences were not due to chance alone. This finding lends some additional support to the suggestion that spondylosis deformans has a heritable genetic component.

This study also found a correlation between body mass and the occurrence of spondylosis deformans in domestic dogs. When non-transport dogs were divided into size-related breed groups the frequency of spondylosis deformans increased with body size. Though small and medium dogs did not differ significantly in the rate of individuals affected with the disease, the rate at which large dogs were affected as compared to small and medium dogs was highly significant suggesting that larger dogs are more prone to the disease. Morgan [20] also found evidence that body mass plays a role in the development of the disease in some dogs. This stresses the need for body size estimation when examining spondylosis deformans in ancient canid remains.

The distribution of osteophytes observed here was similar to those seen in previous studies, though a few key differences were observed. Like Morgan [20], Morgan et al. [22] and Read and Smith [27], the most commonly affected vertebrae were in the mid-thoracic and first few lumbar vertebrae as well as at the L7/sacral joint. However, the degree of cervical involvement in non-transport dogs was higher than in previous studies, and in sled dogs the first three thoracic vertebrae were much more affected than in other studies.

Differences were observed when the data from this study was compared to the rates and distribution of osteophytes in a study of archaeological dogs from Illinois and the southeastern United States [16]. Here, Warren observed minor peaks in the frequency of spondylosis deformans in the first few thoracic vertebrae and in the lower thoracic and lumbar regions [16]. Frequencies near or exceeding 70% were also observed from the twelfth thoracic through fifth lumbar vertebrae, a rate much higher than that observed in any group in the present study.

The sled dogs, like the dogs in Warren’s sample, experienced more involvement in the first few thoracic vertebrae than the non-transport group, but in general, modern canids were more affected in the mid-thoracic vertebrae than Warren’s dogs and less affected in the lower thoracic and lumbar vertebrae. This is especially true for the wolves, which were most affected in the mid-thoracic vertebrae and presented a generally different pattern of distribution than the dogs in Warren’s study.

It is not clear if the differences in the distribution of osteophytes between the dogs described by Warren and our canids occurred because the former dogs were used for carrying packs, as Warren has suggested [16]. In the present study, different patterns of osteophyte distribution were observed in male and female non-transport dogs. Though this does not necessarily explain the pattern of low mid-thoracic involvement in Warren’s dogs, these sex-based differences indicate that factors beyond just habitual activity can affect osteophyte formation and distribution in dogs. While it remains possible that differences in mechanical forces influence the distribution of osteophytes, in the absence of data on spondylosis deformans in known pack dogs, other factors such as sex, breed, age, and size differences known to predispose dogs to marginal osteophytes should also be considered.

**Fig 7:**
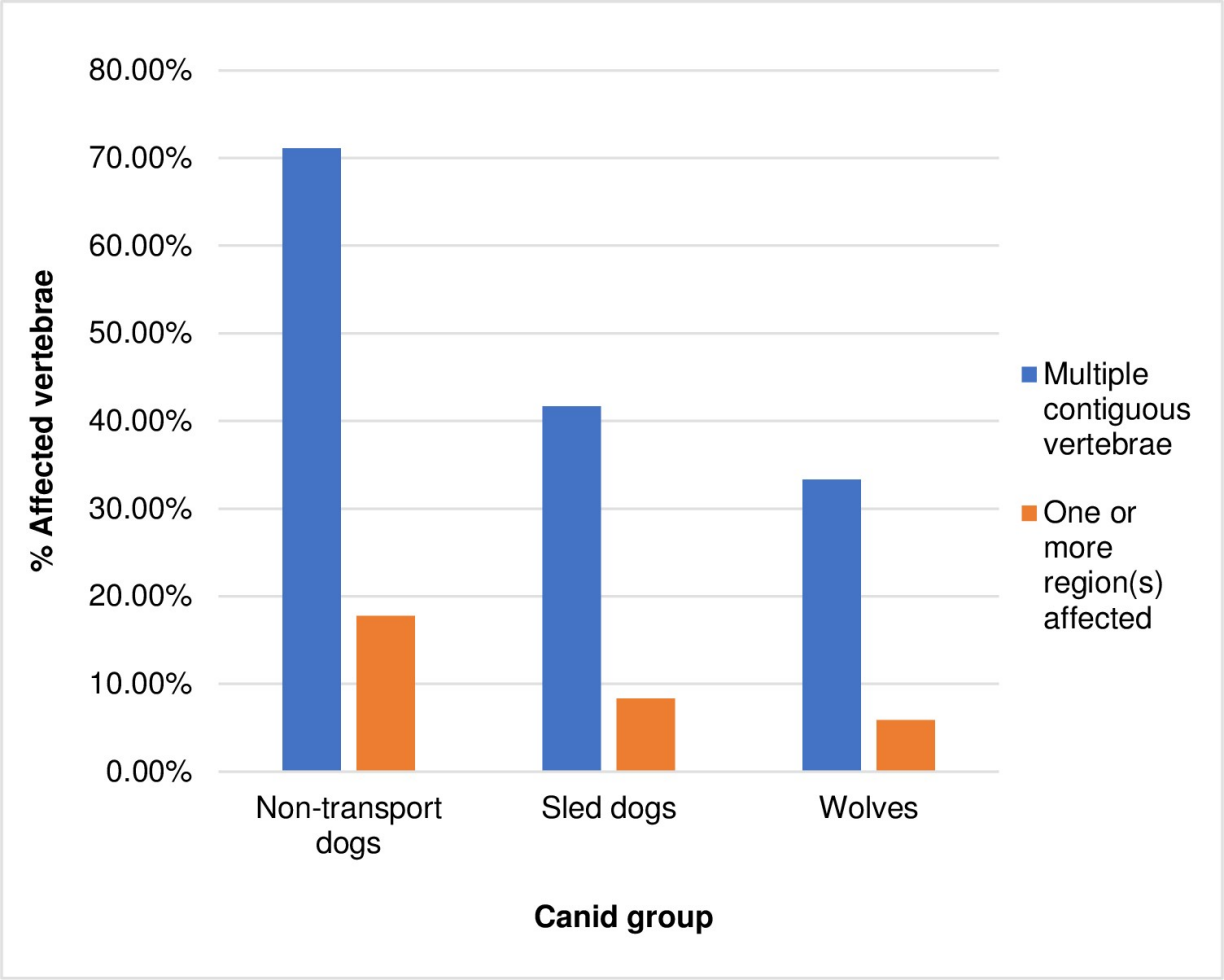
Frequency of multiple contiguous vertebrae (≥3) affected by spondylosis deformans in dogs and wolves.

Both Arnold [10] and Snyder [15] have speculated that in individual dogs, the presence of spondylosis deformans at multiple contiguous vertebrae might be related to transport activities. However, all groups assessed in this study had individuals affected with spondylosis deformans on three or more contiguous vertebrae. Contrary to these earlier studies, this pattern most frequently occurred among non-transport dogs, with 71.11% of individuals with spondylosis deformans having multiple contiguous affected vertebrae compared to a frequency of 41.67% in sled dogs and 33.33% in wolves (Fig 7). While these differences may in part be explained by the generally older age-profile of the non-transport dog group, the average number of contiguous affected vertebrae was similar across groups. Respectively, affected individuals in these groups had an average of 8.89, 7.00, and 9.47 contiguous affected vertebrae. In non-transport dogs 17.78% of individuals with spondylosis deformans had one or more entire vertebral section(s) (i.e.: cervical, thoracic, lumbar) affected by the disease. This occurred less in sled dogs (8.33%) and wolves (5.88%). Based on these results, the presence of spondylosis deformans on three or more contiguous vertebrae, or on entire vertebral sections, is not useful in identifying dogs used in transport activities.

Non-transport dogs in the present study had a significantly higher proportion of endplates affected by the disease than the other canids. This means that individuals affected with the disease showed symptoms in a greater number of vertebrae than individuals in the sled dog or wolf group. It is not clear if these differences reflect the age profiles of each group, or if real differences exist between the analytic groups. In non-transport dogs 20.02% of endplates were affected as compared to 15.89% in sled dogs, and 3.46% in wolves. On average, an individual non-transport dog with the disease had 16.31 endplates affected in their spinal column. Wolves and sled dogs had a similar degree of involvement in affected individuals—affected wolves had an average of 8.80 affected endplates/individual and affected sled dogs had an average of 8.08 affected endplates/individual.

## Conclusions

Systematic analysis of a large sample of canid vertebrae found that dogs and captive wolves are affected by similar rates of spondylosis deformans regardless of their participation in transport activities. Our analysis indicates that several factors including age, sex, breed, and body mass correlate with the development of marginal osteophytes and their distribution in the spines of dogs and wolves. Furthermore, non-transport dogs were affected by marginal osteophytes on a significantly higher number of endplates per individual than either sled dogs or wolves. This suggests that spondylosis deformans might be more visible in archaeological dogs that did not participate in transport activities than in those that did. Differences in the age profiles of our samples might partially explain this pattern, but overall, we find no convincing evidence that transport dogs experienced higher rates of this disease than other canid groups.

Clearly, the presence of spondylosis deformans in dog skeletal remains is not a reliable indicator of their participation in transport activities. Future studies might search for correlations between involvement in transport and degenerative changes in other parts of the skeleton, particularly the limb joints, or changes in limb robusticity and shape related to habitual differences in loading. Given the correlations observed between age and the prevalence and severity of spondylosis deformans, interpretations of this disease (and others that manifest in the skeleton) will benefit from efforts to improve age determination methods for archaeological canid remains. The general relationship between the prevalence of the disease and dog size suggests body mass estimates are necessary datapoints for canid paleopathology. Sex-based differences in the pattern of spondylosis deformans were also observed, pointing for a need to develop better sexing methods for canid skeletal remains.

This study is part of a larger movement within animal paleopathology to develop analytic methods based in empirical research, rather than archaeologist’s interpretations of veterinary and osteological literature [12, 47–54]. By systematically testing skeletal lesions in populations with known life-histories we can improve the quality and accuracy of our interpretations. When these studies are eventually paired with advances in ageing and sexing animal remains, the field of animal paleopathology will make increasingly stronger contributions to the study of the human-animal past.

## Supporting information

S1 Table Specimen data. (XLSX)

S2 Table Dog breeds assessed. (DOCX)

S3 Table Dogs assessed. (DOCX)

S4 Table Wolves assessed. (DOCX)

S5 Table Wolf life history data. (XLSX)

S6 Table Frequency of osteophyte grades in non-transport dog age groups by a) percentage of assessed endplates affected, b) relative frequency of affected endplates by grade.
(DOCX)

S7 Table Frequency of osteophyte grades in sled dog age groups by a) percentage of assessed endplates affected, b) relative frequency of affected endplates by grade.
(DOCX)

S8 Table Frequency of osteophyte grades in wolf age groups by a) percentage of assessed endplates affected, b) relative frequency of affected endplates by grade.
(DOCX)

S1 Fig Severity of spondylosis deformans in non-transport dogs by percentage of endplates affected. (TIF)

S2 Fig Severity of spondylosis deformans in sled dogs by percentage of endplates affected. (TIF)

S3 Fig Severity of spondylosis deformans in wolves by percentage of endplates affected. (TIF)

S4 Fig Distribution of osteophyte grades in all assessed endplates for non-transport dog age groups. (TIF)

S5 Fig Distribution of osteophyte grades in all affected endplates for non-transport dog age groups. (TIF)

S6 Fig Distribution of osteophyte grades in all assessed endplates for sled dog age groups. (TIF)

S7 Fig Distribution of osteophyte grades in all affected endplates for sled dog age groups. (TIF)

S8 Fig Distribution of osteophyte grades in all assessed endplates for wolf age groups. (TIF)

S9 Fig Distribution of osteophyte grades in all affected endplates for wolf age groups. (TIF)
