# Supplementary material for: Spondylosis deformans as an indicator of transport activities in archaeological dogs: A systematic evaluation of current methods for assessing archaeological specimens
Source: PLoS One. 2019 Apr 17;14(4):e0214575. doi: 10.1371/journal.pone.0214575 (PMC6469781; doi:10.1371/journal.pone.0214575)
Supplement: S2 Table — (DOCX) [file pone.0214575.s002.docx]

# **S2 Table. Dog breeds assessed.**

| \| English name \| Other names \| Total \| Size group \| \| --- \| --- \| --- \| --- \| \| Afghan Hound \| Afghanischeer Windhund \| 1 \| large \| \| Appenzeller Sennenhund \| Appenzeller Sennenhund \| 2 \| medium \| \| Australian Terrier \| Australian Terrier \| 1 \| small \| \| Austrian Black and Tan Hound \| Österreichische Bracke (Brandl) \| 1 \| medium \| \| Belgian Tervuren \| Tervueren \| 1 \| medium \| \| Bernese Mountain Dog \| Berner Sennenhund \| 2 \| large \| \| Border Terrier \| Border Terrier \| 2 \| small \| \| Borzoi \| Russian Wolfhound \| 4 \| large \| \| Boston Terrier \| Boston Terrier \| 1 \| small \| \| Boxer \| Boxer \| 1 \| medium \| \| Briard \| Berger de Brie \| 1 \| medium \| \| Bullmastiff \| Bullmastiff \| 1 \| large \| \| Cairn Terrier \| Cairn Terrier \| 1 \| small \| \| Cavalier king Charles Spaniel \| King Charles Spaniel Cavalier \| 2 \| small \| \| Chihuahua \| Chihuahua \| 7 \| small \| \| Dachshund \| Dachshund \| 2 \| small \| \| Deutsch Stichelhaar \| Vorstenhund, Dt. Stichelhaar \| 1 \| medium \| \| Eurasier \| Eurasier \| 2 \| small \| \| German Shepherd \| German Shepherd \| 1 \| large \| \| German Spaniel \| Deutscher Wachtelhund \| 2 \| medium \| \| Gordon Setter \| Gordon Setter \| 1 \| large \| \| Great Dane \| Deutsche Dogge \| 1 \| large \| \| Greater Swiss Mountain Dog \| Grosser Schweizer Sennenhund \| 1 \| large \| \| Greyhound \| Greyhound \| 4 \| large \| \| Havanese \| Havaneser \| 1 \| small \| \| Hovawart \| Hovawart \| 3 \| medium \| \| Irish Setter \| Irish Setter \| 1 \| large \| \| Irish Wolfhound \| Irish Wolfhound \| 6 \| large \| \| Italian Greyhound \| Italian Greyhound \| 2 \| small \| \| Leonberger \| Leonberger \| 1 \| large \| \| Mastiff \| Mastiff \| 1 \| large \| \| Miniature Pinscher \| Zwergpinscher \| 1 \| small \| \| New Foundland \| New Foundland \| 2 \| large \| \| Norwich Terrier \| Norwich Terrier \| 2 \| small \| \| Nova Scotia Duck Tolling Retriever \| Nova Scotia Duck Tolling Retriever \| 1 \| medium \| \| Old English Sheepdog \| Bobtail \| 1 \| medium \| \| Otterhound \| Pure bred otterhound \| 1 \| large \| \| Pembroke Welsh Corgi \| Welsch Corgi Pembroke \| 1 \| medium \| \| Perro de Presa Canario \| Dogo Canario \| 1 \| large \| \| Peruvian Inca Orchid (Peruvian Hairless Dog) \| Peruanischer nackthund (Perro sin Pelo del Peru) \| 1 \| small \| \| Pit bull \| Pit bull \| 1 \| medium \| \| Pug \| Mops \| 1 \| small \| \| Rhodesian Ridgeback \| Rhodesian Ridgeback \| 3 \| medium \| \| Scottish Deer Hound \| Scottish Deer Hound \| 1 \| large \| \| Scottish Terrier \| Scottish Terrier \| 2 \| small \| \| Siberian Husky \| Siberian Husky \| 1 \| medium \| \| Skye Terrier \| Skye Terrier \| 1 \| medium \| \| Slovensky Cuvac \| Slovensky Tschuwatsch \| 2 \| medium \| \| Spanish Bulldog \| Alano Espanol \| 1 \| large \| \| Springer Spaniel \| Springer Spaniel \| 1 \| large \| \| St. Bernard \| St. Bernhardshund \| 12 \| large \| \| Swiss Hound \| Schwyzer Laufhund \| 1 \| medium \| \| Tibetan mastiff \| Tibetan mastiff \| 1 \| large \| \| Toy poodle \| Toy poodle \| 1 \| small \| \| Unknown \|  \| 19 \| unknown \| \| Vizsla \| Vizsla \| 1 \| medium \| \| Welsh Springer Spaniel \| English Springer Spaniel \| 1 \| medium \| \| White Swiss Shepherd Dog \| Berger Blanc Suisse \| 4 \| large \| \| Xoloitzcuintli \| Mexikanischer Nackthund (Xoloitzcuintle) \| 3 \| medium \| \| Total \|  \| **125** \|  \| |  |  |  |
| --- | --- | --- | --- | --- | --- | --- | --- | --- | --- | --- | --- | --- | --- | --- | --- | --- | --- | --- | --- | --- | --- | --- | --- | --- | --- | --- | --- | --- | --- | --- | --- | --- | --- | --- | --- | --- | --- | --- | --- | --- | --- | --- | --- | --- | --- | --- | --- | --- | --- | --- | --- | --- | --- | --- | --- | --- | --- | --- | --- | --- | --- | --- | --- | --- | --- | --- | --- | --- | --- | --- | --- | --- | --- | --- | --- | --- | --- | --- | --- | --- | --- | --- | --- | --- | --- | --- | --- | --- | --- | --- | --- | --- | --- | --- | --- | --- | --- | --- | --- | --- | --- | --- | --- | --- | --- | --- | --- | --- | --- | --- | --- | --- | --- | --- | --- | --- | --- | --- | --- | --- | --- | --- | --- | --- | --- | --- | --- | --- | --- | --- | --- | --- | --- | --- | --- | --- | --- | --- | --- | --- | --- | --- | --- | --- | --- | --- | --- | --- | --- | --- | --- | --- | --- | --- | --- | --- | --- | --- | --- | --- | --- | --- | --- | --- | --- | --- | --- | --- | --- | --- | --- | --- | --- | --- | --- | --- | --- | --- | --- | --- | --- | --- | --- | --- | --- | --- | --- | --- | --- | --- | --- | --- | --- | --- | --- | --- | --- | --- | --- | --- | --- | --- | --- | --- | --- | --- | --- | --- | --- | --- | --- | --- | --- | --- | --- | --- | --- | --- | --- | --- | --- | --- | --- | --- | --- | --- | --- | --- | --- | --- | --- | --- | --- | --- | --- | --- | --- | --- | --- | --- | --- | --- | --- | --- | --- | --- | --- |
