# Supplementary material for: Spondylosis deformans as an indicator of transport activities in archaeological dogs: A systematic evaluation of current methods for assessing archaeological specimens
Source: PLoS One. 2019 Apr 17;14(4):e0214575. doi: 10.1371/journal.pone.0214575 (PMC6469781; doi:10.1371/journal.pone.0214575)
Supplement: S3 Table — (DOCX) [file pone.0214575.s003.docx]

**S3 Table. Dogs assessed.**

|  | All groups | Non-transport dogs | Wild dingoes | Captive dingoes | Sled dogs |
| --- | --- | --- | --- | --- | --- |
| Female | 61 | 54 | 2 | 1 | 4 |
| Male | 69 | 55 | 2 | 5 | 7 |
| Unknown | 25 | 16 | 1 | 0 | 8 |
| Adult | 149 | 121 | 4 | 5 | 19 |
| Juvenile | 6 | 4 | 1 | 1 | 0 |
| Total | **155** | **125** | **5** | **6** | **19** |
