# Supplementary material for: Spondylosis deformans as an indicator of transport activities in archaeological dogs: A systematic evaluation of current methods for assessing archaeological specimens
Source: PLoS One. 2019 Apr 17;14(4):e0214575. doi: 10.1371/journal.pone.0214575 (PMC6469781; doi:10.1371/journal.pone.0214575)
Supplement: S4 Table — (DOCX) [file pone.0214575.s004.docx]

**S4 Table.** **Wolves Assessed**

|  | All | Wild | Captive | Inbred | Non-Inbred |
| --- | --- | --- | --- | --- | --- |
| Female | 103 | 99 | 4 | 35 | 68 |
| Male | 124 | 117 | 7 | 44 | 80 |
| Unknown | 14 | 12 | 2 | 2 | 12 |
| Adult | 167 | 155 | 12 | 58 | 109 |
| Juvenile | 74 | 73 | 1 | 23 | 51 |
| Total | **241** | **228** | **13** | **81** | **160** |
