# Supplementary material for: Spondylosis deformans as an indicator of transport activities in archaeological dogs: A systematic evaluation of current methods for assessing archaeological specimens
Source: PLoS One. 2019 Apr 17;14(4):e0214575. doi: 10.1371/journal.pone.0214575 (PMC6469781; doi:10.1371/journal.pone.0214575)
Supplement: S6 Table — Frequency of osteophyte grades in non-transport dog age groups by a) percentage of assessed endplates affected, b) relative frequency of affected endplates by grade. (DOCX) [file pone.0214575.s006.docx]

**S6 Table.** **Frequency of osteophyte grades in non-transport dog age groups by a) percentage of assessed endplates affected, b) relative frequency of affected endplates by grade.**

| 3a. |  |  |  |  |
| --- | --- | --- | --- | --- |
| Age Group | **Assessed Endplates** | **Grade 1** | **Grade 2** | **Grade 3** |
| 0-2 | 270 | 7(2.59) | 5(1.85) | 0(0.00) |
| 3-5 | 432 | 20(4.63) | 3(0.69) | 1(0.23) |
| 6-8 | 486 | 48(9.88) | 8(1.65) | 2(0.41) |
| 9-11 | 1294 | 288(22.26) | 157(12.13) | 22(1.70) |
| 12-14 | 1024 | 225(21.97) | 117(11.43) | 0(0.00) |
| 15-17 | 644 | 155(24.07) | 70(10.87) | 5(0.78) |
| Total | **4150** | **743(17.90)** | **360(8.67)** | **30(0.72)** |

| 3b. |  |  |  |  |
| --- | --- | --- | --- | --- |
| Age Group | **Affected Endplates** | **Grade 1** | **Grade 2** | **Grade 3** |
| 0-2 | 12 | 7(58.33) | 5(41.67) | 0(0.00) |
| 3-5 | 24 | 20(83.33) | 3(12.5) | 1(4.17) |
| 6-8 | 58 | 48(82.76) | 8(13.79) | 2(3.45) |
| 9-11 | 467 | 288(61.67) | 157(33.62) | 22(4.71) |
| 12-14 | 342 | 225(65.79) | 117(34.21) | 0(0.00) |
| 15-17 | 230 | 155(67.39) | 70(30.43) | 5(2.17) |
| Total | **1133** | **743(65.58)** | **360(31.77)** | **30(2.65)** |
