# Supplementary material for: Spondylosis deformans as an indicator of transport activities in archaeological dogs: A systematic evaluation of current methods for assessing archaeological specimens
Source: PLoS One. 2019 Apr 17;14(4):e0214575. doi: 10.1371/journal.pone.0214575 (PMC6469781; doi:10.1371/journal.pone.0214575)
Supplement: S7 Table — Frequency of osteophyte grades in sled dog age groups by a) percentage of assessed endplates affected, b) relative frequency of affected endplates by grade. (DOCX) [file pone.0214575.s007.docx]

**S7 Table.** **Frequency of osteophyte grades in sled dog age groups by a) percentage of assessed endplates affected, b) relative frequency of affected endplates by grade.**

| 4a. |  |  |  |  |
| --- | --- | --- | --- | --- |
| Age Group | **Assessed Endplates** | **Grade 1** | **Grade 2** | **Grade 3** |
| 3-5 | 108 | 4(3.70) | 0(0.0) | 0(0.00) |
| 9-11 | 324 | 21(6.48) | 7(2.16) | 0(0.00) |
| 12-14 | 108 | 42(38.89) | 5(4.63) | 0(0.00) |
| Total | 540 | 67(12.41) | 12(22.22) | 0(0.00) |

| 4b. |  |  |  |  |
| --- | --- | --- | --- | --- |
| Age Group | **Affected Endplates** | **Grade 1** | **Grade 2** | **Grade 3** |
| 3-5 | 48 | 4(100.00) | 0(0.0) | 0(0.00) |
| 9-11 | 28 | 21(75.00) | 7(25.00) | 0(0.00) |
| 12-14 | 47 | 42(89.36) | 5(10.64) | 0(0.00) |
| Total | 79 | 67(84.81) | 12(15.19) | 0(0.00) |
