# Supplementary material for: Spondylosis deformans as an indicator of transport activities in archaeological dogs: A systematic evaluation of current methods for assessing archaeological specimens
Source: PLoS One. 2019 Apr 17;14(4):e0214575. doi: 10.1371/journal.pone.0214575 (PMC6469781; doi:10.1371/journal.pone.0214575)
Supplement: S8 Table — Frequency of osteophyte grades in wolf age groups by a) percentage of assessed endplates affected, b) relative frequency of affected endplates by grade. (DOCX) [file pone.0214575.s008.docx]

**S8 Table.** **Frequency of osteophyte grades in wolf age groups by a) percentage of assessed endplates affected, b) relative frequency of affected endplates by grade.**

| 5a. |  |  |  |  |
| --- | --- | --- | --- | --- |
| Age Group | **Assessed Endplates** | **Grade 1** | **Grade 2** | **Grade 3** |
| 0-2 | 2592 | 10(0.39) | 6(0.23) | 2(0.08) |
| 3-5 | 702 | 9(1.28) | 4(0.57) | (0.00) |
| 6-8 | 322 | 31(9.63) | 10(3.11) | (0.00) |
| 9-11 | 160 | 38(23.75) | 30(18.75) | 2(1.25) |
| 12-14 | 54 | 16(29.63) | 21(38.89) | 8(14.81) |
| Total | 3830 | 104(2.72) | 71(1.85) | 12(0.31) |

| 5b. |  |  |  |  |
| --- | --- | --- | --- | --- |
| Age Group | **Affected**  **Endplates** | **Grade 1** | **Grade 2** | **Grade 3** |
| 0-2 | 18 | 10(55.56) | 6(33.33) | 2(11.11) |
| 3-5 | 13 | 9(69.23) | 4(30.77) | 0(0.00) |
| 6-8 | 41 | 31(69.23) | 4(30.77) | 0(0.00) |
| 9-11 | 70 | 38(54.29) | 30(42.86) | 2(2.86) |
| 12-14 | 45 | 16(35.56) | 21(46.67) | 8(17.78) |
| Total | 187 | 104(55.61) | 71(37.97) | 12(6.42) |
