# Supplementary figures and images for: Spondylosis deformans as an indicator of transport activities in archaeological dogs: A systematic evaluation of current methods for assessing archaeological specimens
Source: PLoS One. 2019 Apr 17;14(4):e0214575. doi: 10.1371/journal.pone.0214575 (PMC6469781; doi:10.1371/journal.pone.0214575)

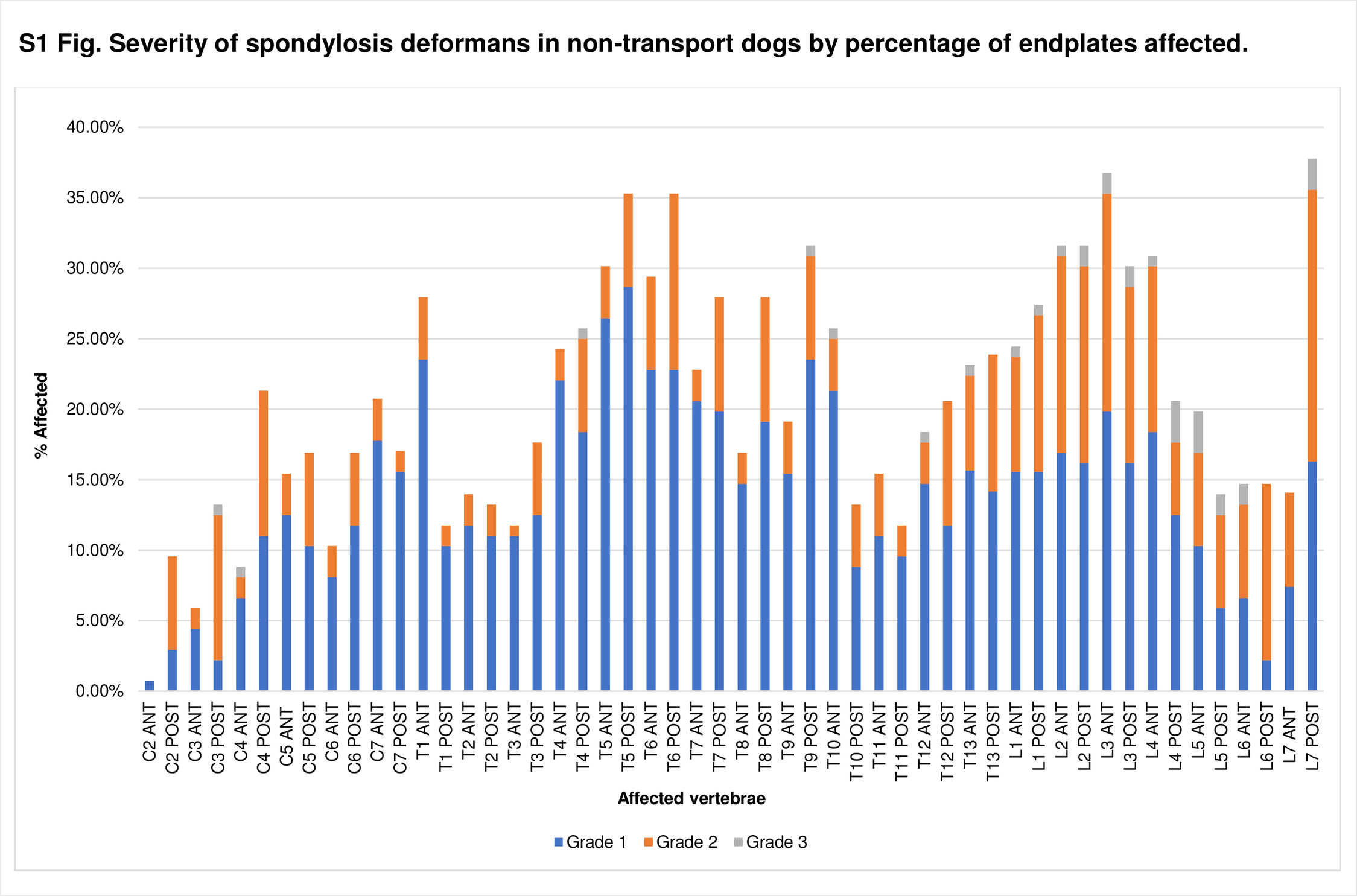

Supplement: S1 Fig — (TIF) [file pone.0214575.s009.tif]

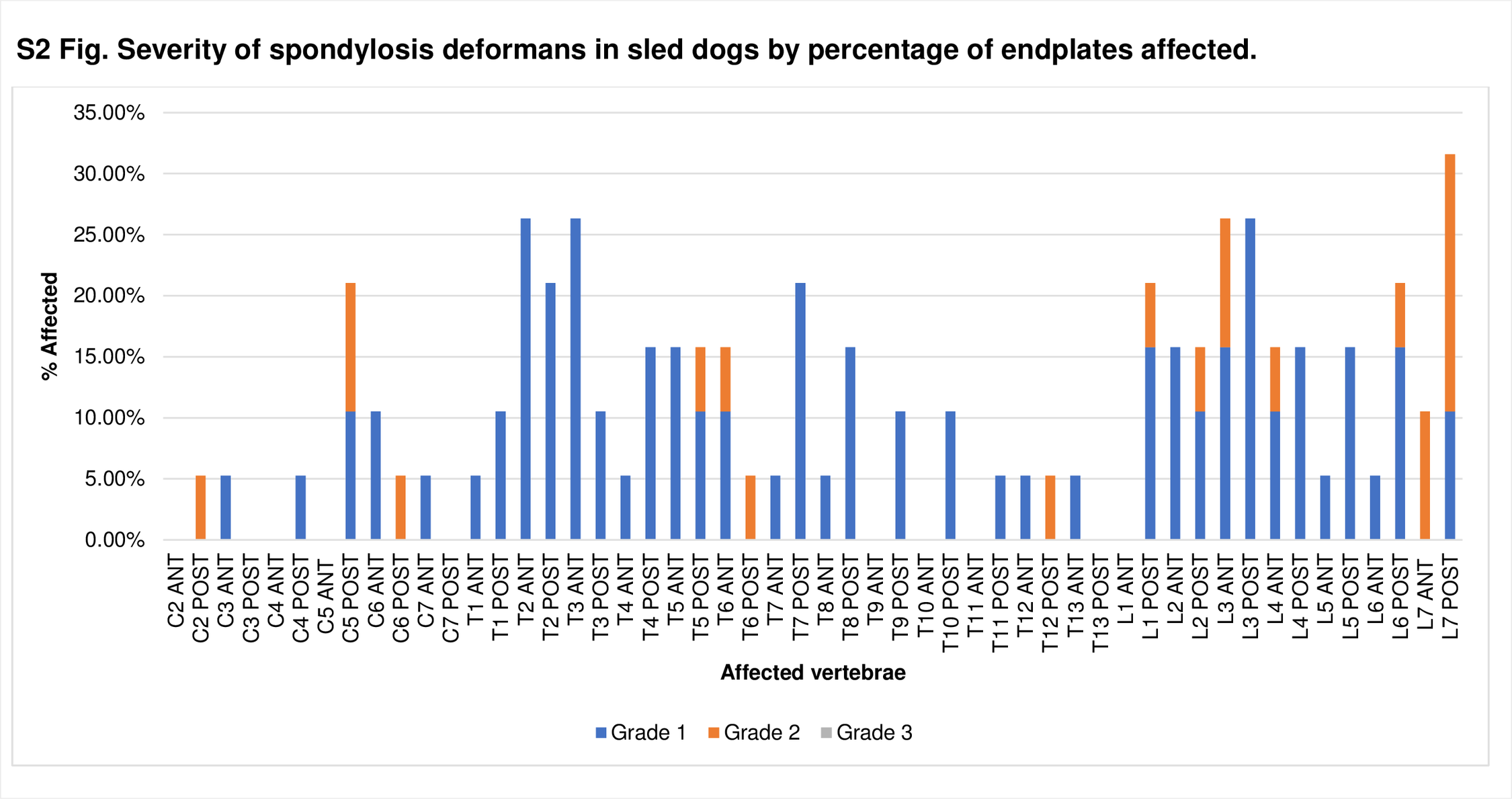

Supplement: S2 Fig — (TIF) [file pone.0214575.s010.tif]

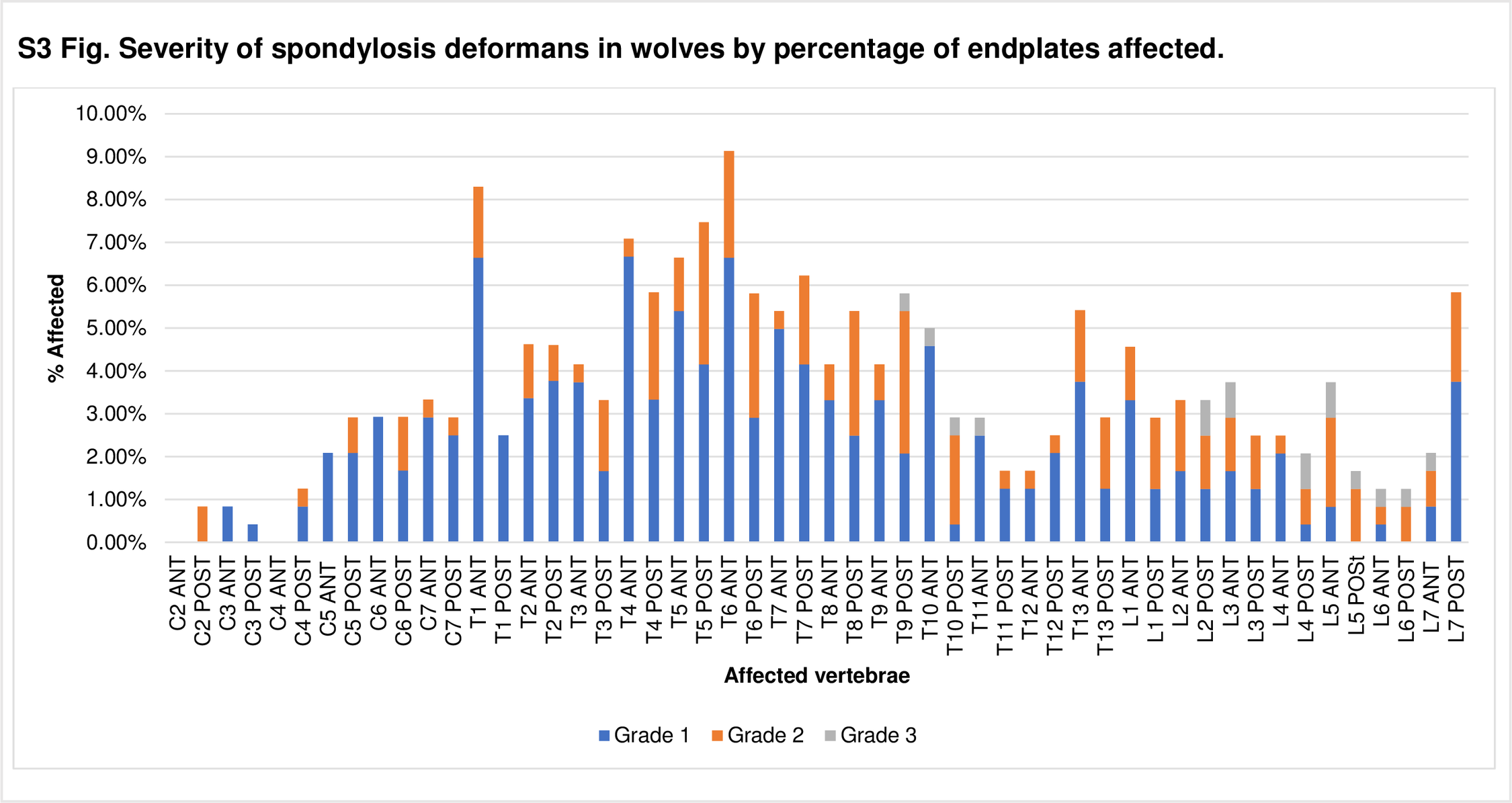

Supplement: S3 Fig — (TIF) [file pone.0214575.s011.tif]

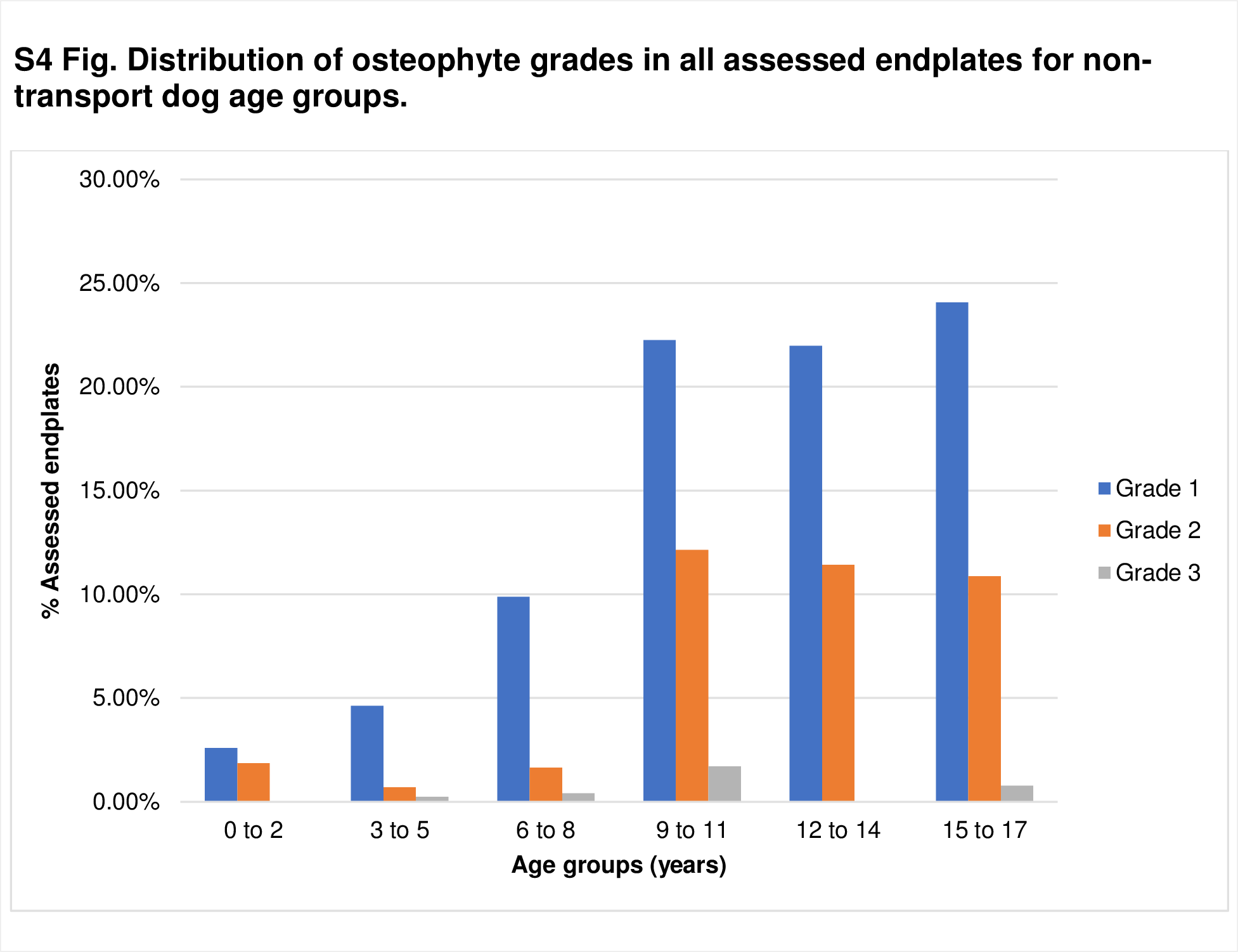

Supplement: S4 Fig — (TIF) [file pone.0214575.s012.tif]

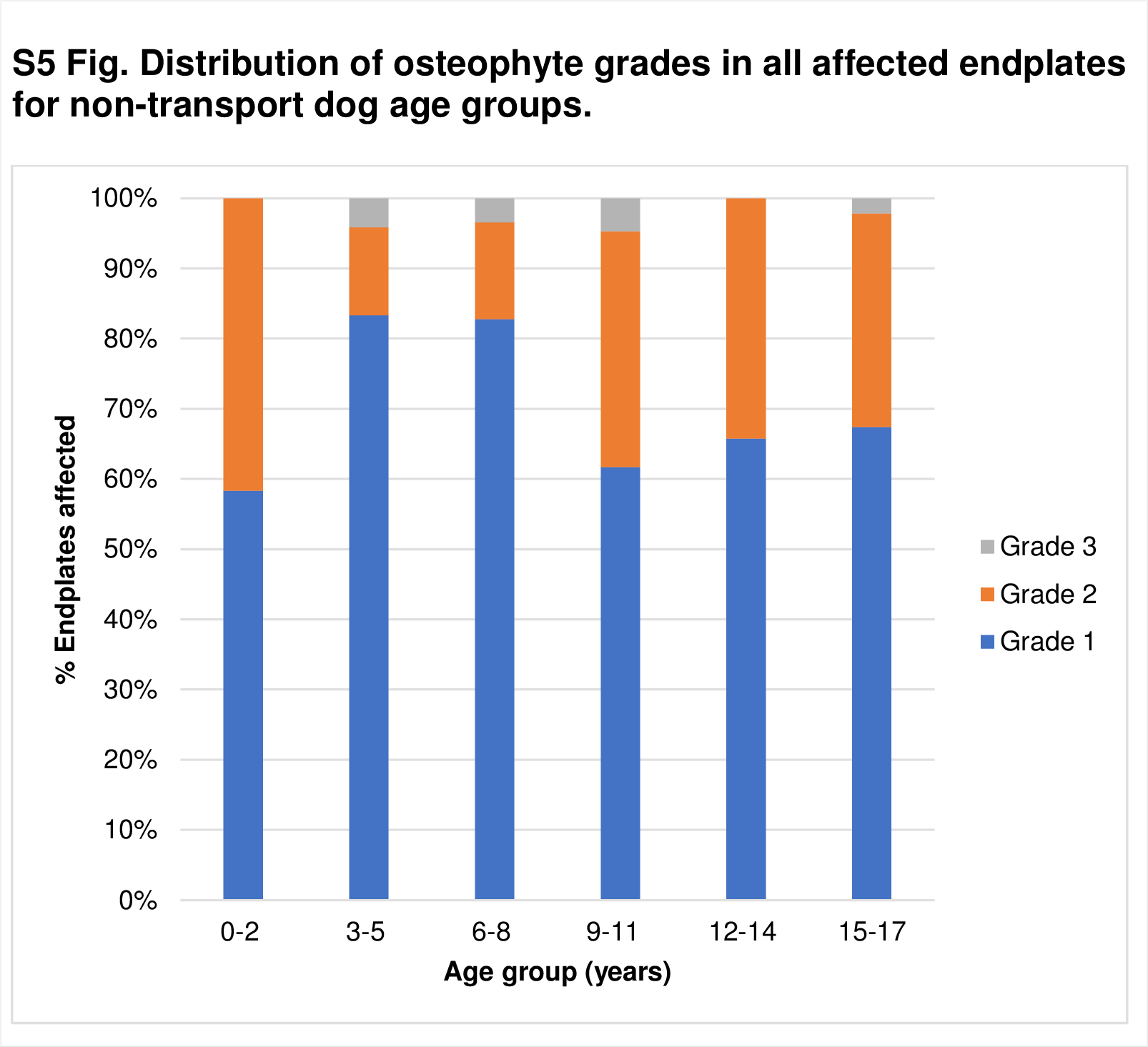

Supplement: S5 Fig — (TIF) [file pone.0214575.s013.tif]

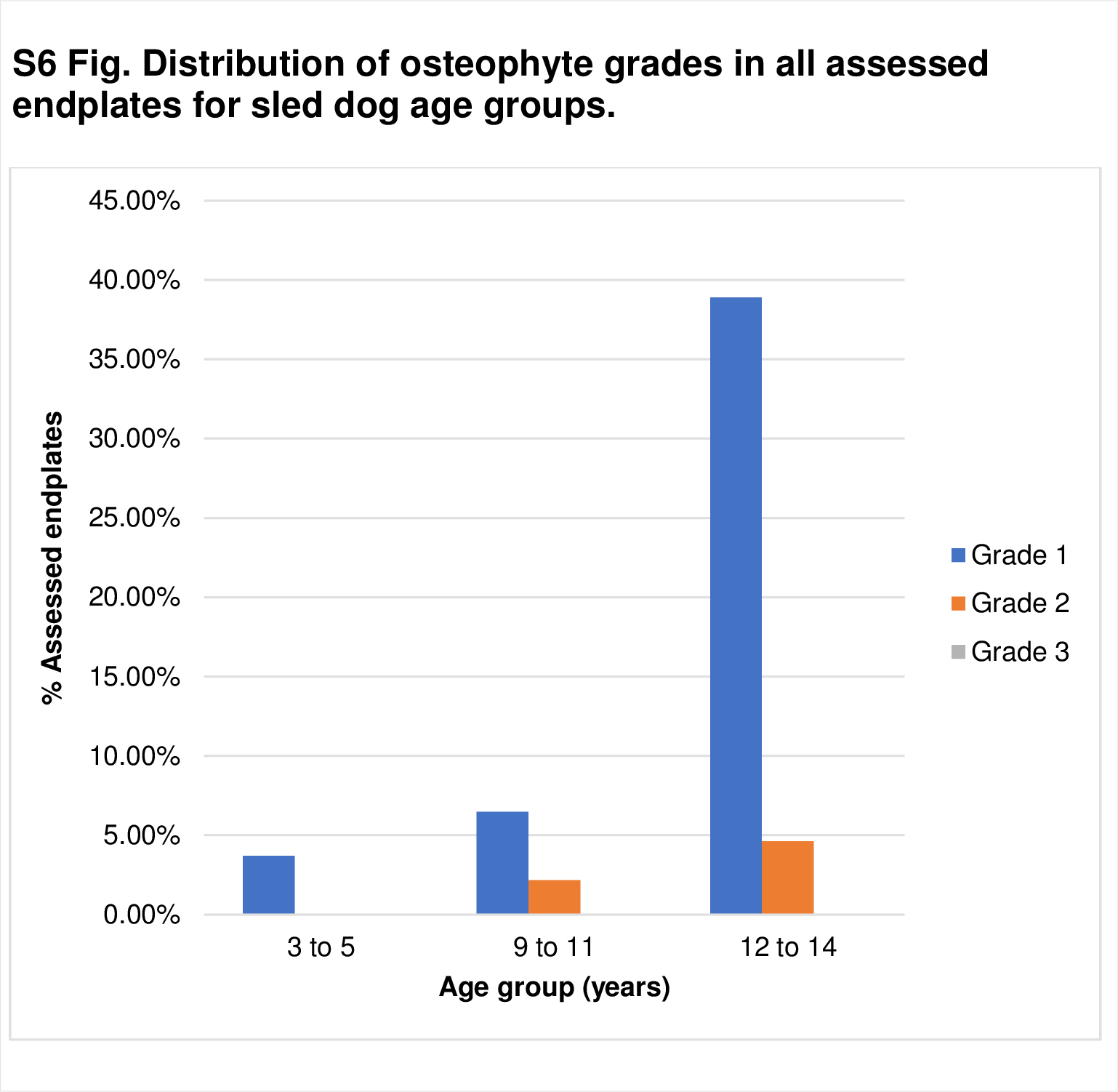

Supplement: S6 Fig — (TIF) [file pone.0214575.s014.tif]

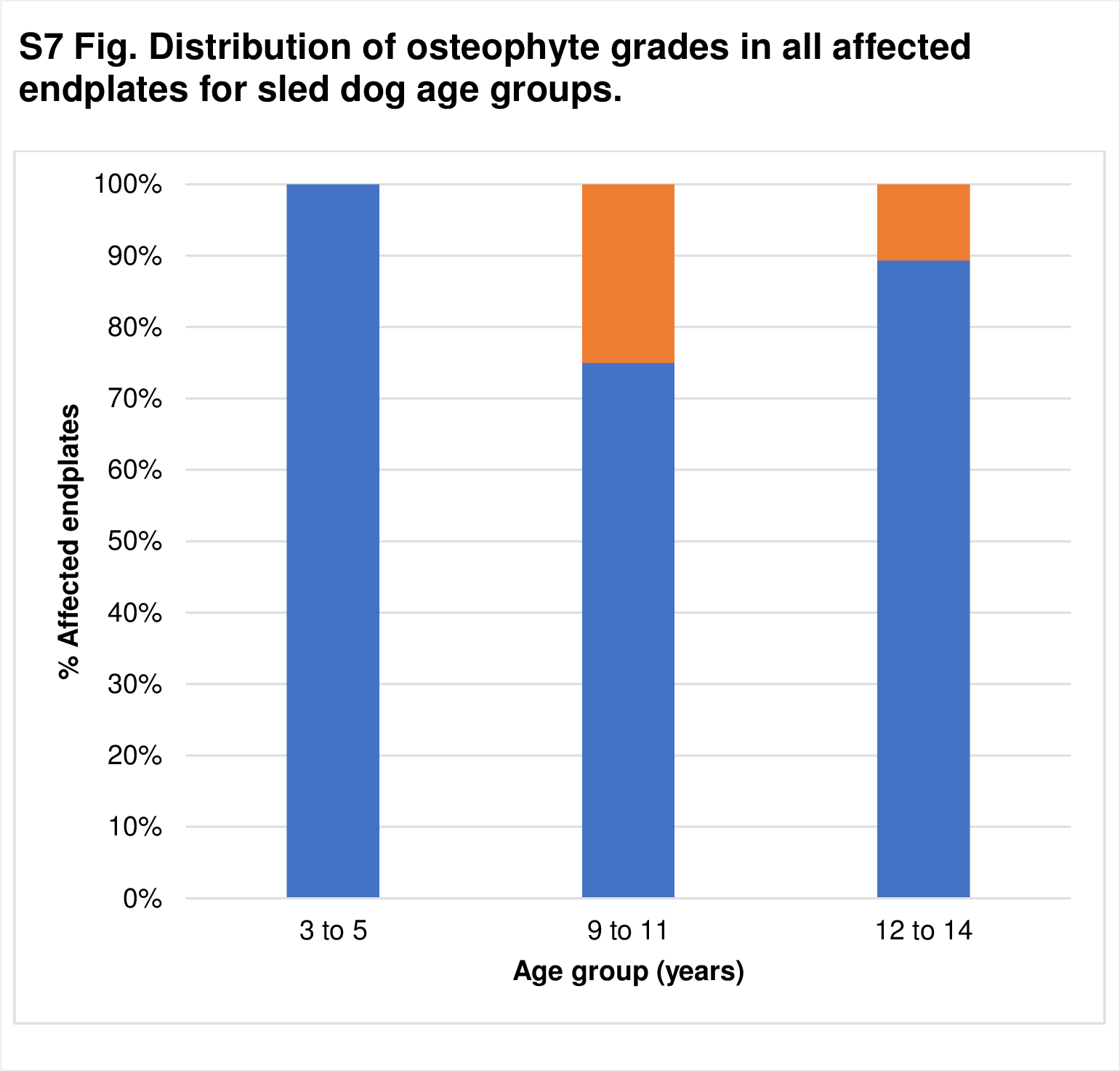

Supplement: S7 Fig — (TIF) [file pone.0214575.s015.tif]

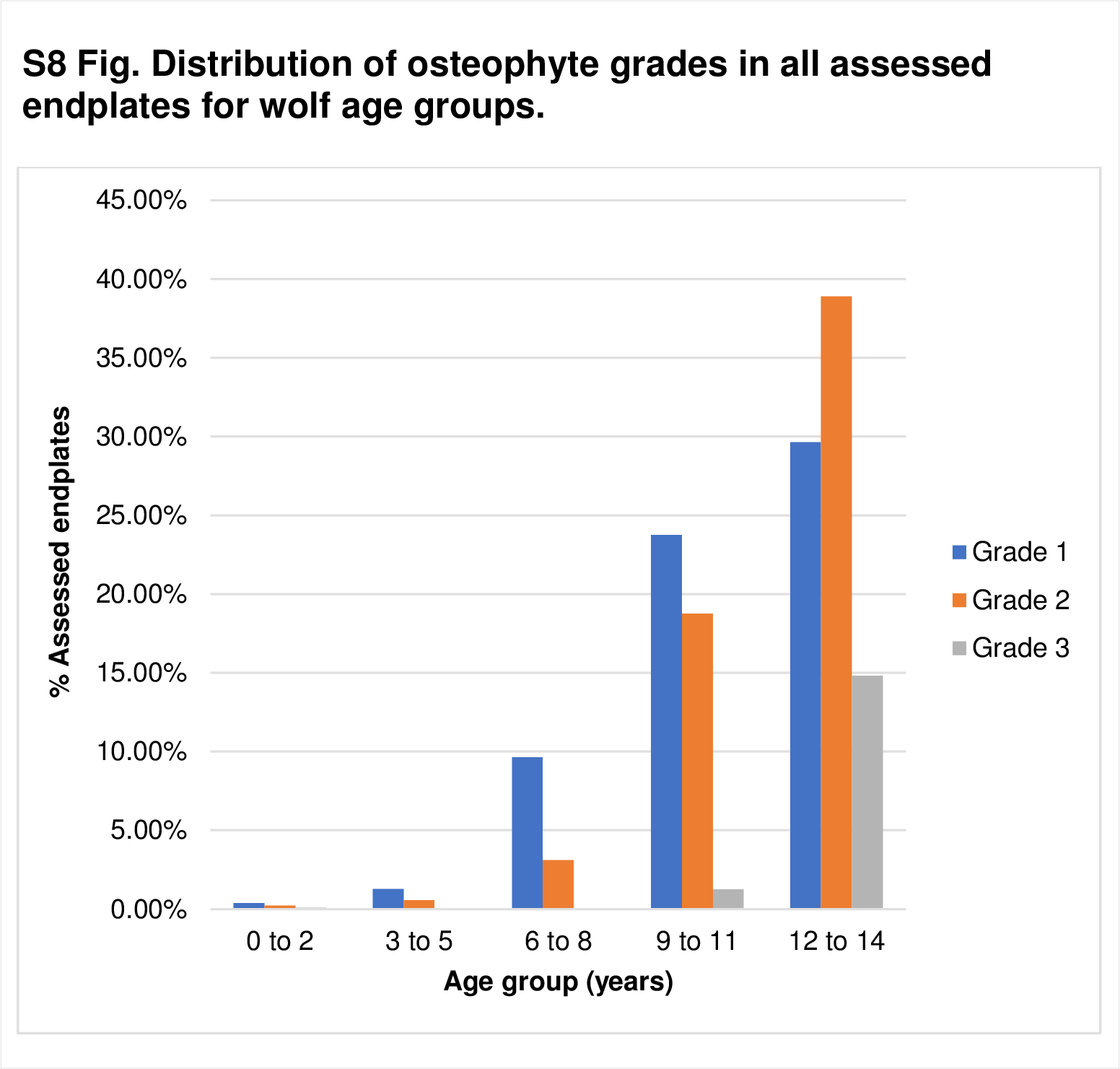

Supplement: S8 Fig — (TIF) [file pone.0214575.s016.tif]

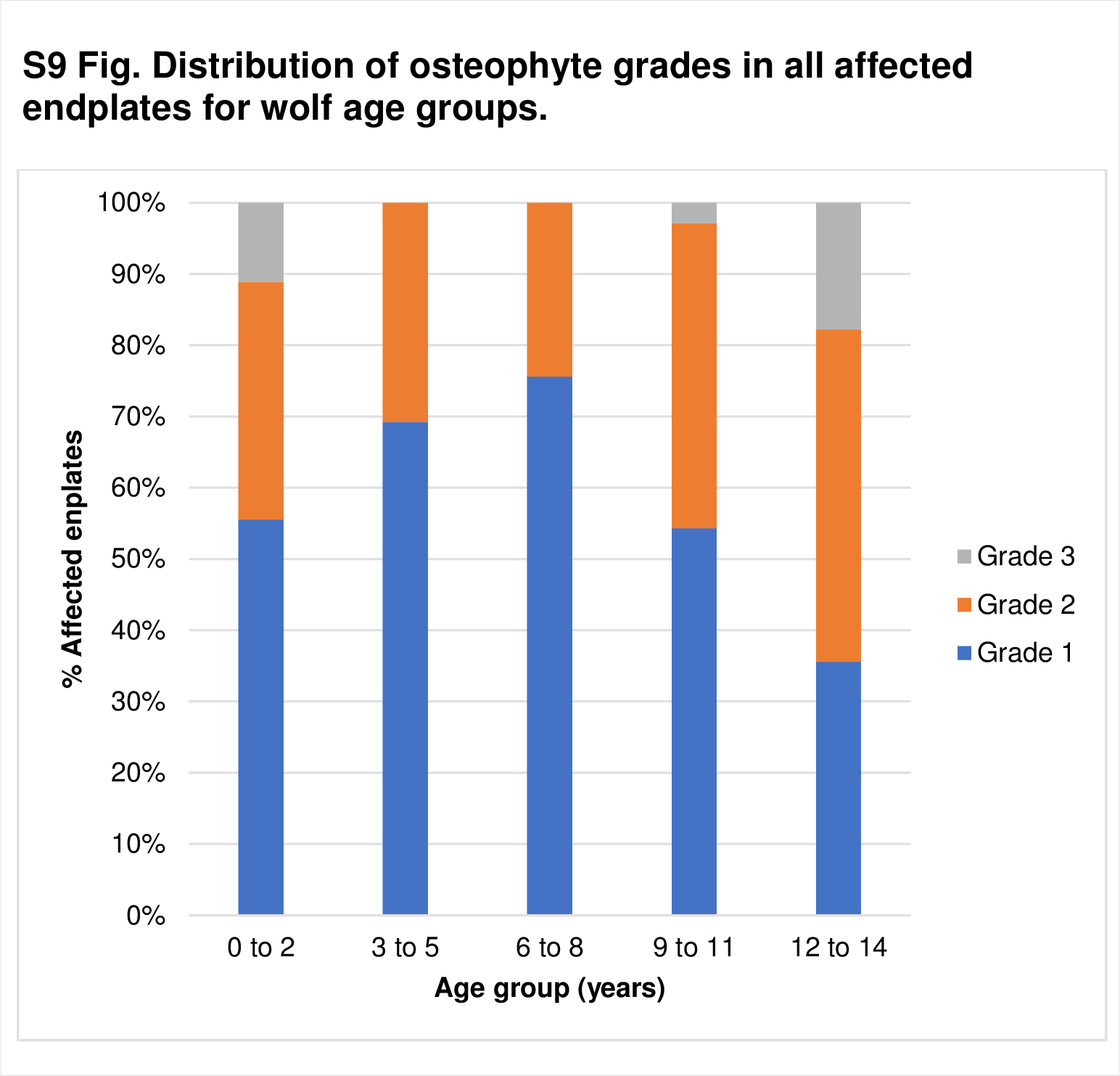

Supplement: S9 Fig — (TIF) [file pone.0214575.s017.tif]
